# Curcumin in the treatment of inflammation and oxidative stress responses in traumatic brain injury: a systematic review and meta-analysis

**DOI:** 10.3389/fneur.2024.1380353

**Published:** 2024-05-10

**Authors:** Jinfeng Guo, Zhengjie Li, Yun Yao, Lei Fang, Mingdi Yu, Zuhui Wang

**Affiliations:** ^1^The Nursing Department of Anhui College of Traditional Chinese Medicine, Wuhu, Anhui, China; ^2^The Outpatient and Emergency Department of Wuhu Hospital of Traditional Chinese Medicine, Wuhu, Anhui, China

**Keywords:** curcumin, traumatic brain injury, TBI, inflammation, oxidative stress

## Abstract

**Background and aim:**

Traumatic brain injury (TBI), a leading cause of high morbidity and mortality, represents a significant global public health challenge. Currently, no effective treatment for TBI exists. Curcumin, an active compound extracted from the root of *Curcuma longa*, has demonstrated neuroprotective properties both *in vitro* and *in vivo*. Notably, it has shown potential in reducing oxidative stress and inflammation and enhancing redox balance. This paper conducts a systematic review and meta-analysis to explore curcumin’s role in TBI animal models extensively. The findings offer valuable insights for future human clinical trials evaluating curcumin as a therapeutic supplement or nutraceutical in TBI management.

**Methods:**

Comprehensive literature searches were conducted across MEDLINE, Embase, Cochrane, Web of Science, and Google Scholar databases. These searches aimed to identify relevant manuscripts in all languages, utilizing the keywords “curcumin” and “traumatic brain injury.”

**Results:**

The final quantitative analysis included 18 eligible articles corresponding to animal studies. The analysis revealed that curcumin significantly reduced inflammatory cytokines, including IL-1β (*p* = 0.000), IL-6 (*p* = 0.002), and TNF-α (*p* = 0.000), across various concentrations, time points, and administration routes. Additionally, curcumin markedly enhanced the activity of oxidative stress markers such as SOD (*p* = 0.000), Sir2 (*p* = 0.000), GPx (*p* = 0.000), and Nrf2 (*p* = 0.000), while reducing MDA (*p* = 0.000), 4-HNE (*p* = 0.001), and oxyprotein levels (*p* = 0.024). Furthermore, curcumin improved cerebral edema (*p* = 0.000) and upregulated neuroprotective factors like synapsin I (*p* = 0.019), BDNF (*p* = 0.000), and CREB (*p* = 0.000), without reducing mNSS (*p* = 0.144). About autophagy and apoptosis, curcumin increased the activity of Beclin-1 (*p* = 0.000) and Bcl-2 (*p* = 0.000), while decreasing caspase-3 (*p* = 0.000), the apoptosis index (*p* = 0.000), and P62 (*p* = 0.002).

**Conclusion:**

Curcumin supplementation positively affects traumatic brain injury (TBI) by alleviating oxidative stress and inflammatory responses and promoting neuroprotection. It holds potential as a therapeutic agent for human TBI. However, this conclusion necessitates further substantiation through high-quality literature and additional randomized controlled trials (RCTs).

**Systematic Review Registration:**

https://www.crd.york.ac.uk/prospero/. The registration number of PROSPERO: CRD42023452685.

## 1 Introduction

Traumatic brain injury (TBI) has a high morbidity and mortality rate, remaining a leading cause of disability and health complications globally (1). An estimated 10 million individuals are either hospitalized or die annually due to TBI, and approximately 57 million people have experienced such an injury (2). Traumatic brain injury (TBI) is considered to be a biphasic injury with both primary and secondary injury properties. Secondary injuries occur within hours to days of the initial injury. It may be caused by inflammation, oxidative stress, calcium homeostasis, disruption of the blood–brain barrier, etc. Secondary injury exacerbates brain damage after TBI (3). Due to improvements in pre-hospital and neurological intensive care, current treatment of TBI focuses on avoiding or mitigating secondary injury processes rather than repairing damage caused by the primary injury (4). Curcumin, the primary ingredient of turmeric, is recognized by the U.S. Food and Drug Administration as safe for human use (5, 6). Its chemical structure is 2α, β-unsaturated β-dione, with the chemical name (E, E) -1,7-β (4-hydroxy-3-methoxyphenyl) -1, 6-heptane-3, 5-dione, and a melting point of 179°C–183°C (7). Research (8) indicates that curcumin can be rapidly absorbed into the bloodstream and brain, substantially ameliorating secondary brain injuries caused by TBI, including cerebral edema and oxidative stress. A randomized controlled trial (RCT) by Zahedi et al. (9) involving 62 adult TBI patients in ICU settings revealed that short-term curcumin supplementation improved inflammatory biomarkers and clinical outcomes, alongside nutritional status, but had no impact on oxidative stress markers. However, studies on the effects of curcumin and its derivatives on human TBI are limited. Eghbaliferiz et al. (10) reported that curcumin possesses anti-tumor and neuroprotective properties. Despite these findings, no comprehensive systematic analysis of all available data has been conducted. Therefore, this study aims to perform a systematic review and meta-analysis of animal studies to inform the design of future human clinical trials using curcumin as a supplement or nutrient. This research specifically focuses on evaluating the anti-inflammatory and antioxidant effects of curcumin in animal models of TBI, with a particular emphasis on changes in inflammatory factors such as interleukin-1β (IL-1β), interleukin-6 (IL-6), tumor necrosis factor-α (TNF-α), and oxidative stress markers including superoxide dismutase (SOD), silent information regulator 2 (Sir2), malondialdehyde (MDA), glutathione peroxidase (GPx), NF-E2-related factor (Nrf2), and others.

## 2 Materials and methods

### 2.1 Search strategy

A comprehensive systematic search was conducted for manuscripts in various languages, published from the inception of each database until January 2023. This search utilized five databases: Embase, Web of Science, Cochrane, MEDLINE, and Google Scholar. The search strategy was formulated based on the PICOS framework: (P) Population: animals with TBI; (I) Intervention: curcumin; (C) Comparator: control groups comprising TBI animals receiving either conventional therapy or a blank control; (O) Outcomes: changes in inflammatory and oxidative stress factors post-TBI; (S) Study Type: RCTs. The specific search strategy is detailed in Table 1 (PubMed is used as an example).

**Table 1:** Search strategy on PubMed.

Database	Search string
PubMed	((“Brain Injuries, Traumatic”[Mesh]) OR (((((((((((((((Brain Injury, Traumatic) OR (Traumatic Brain Injuries)) OR (Trauma, Brain)) OR (Brain Trauma)) OR (Brain Traumas)) OR (Traumas, Brain)) OR (TBI (Traumatic Brain Injury))) OR (Encephalopathy, Traumatic)) OR (Encephalopathies, Traumatic)) OR (Traumatic Encephalopathies)) OR (Injury, Brain, Traumatic)) OR (Traumatic Encephalopathy)) OR (TBIs (Traumatic Brain Injuries))) OR (TBI (Traumatic Brain Injuries))) OR (Traumatic Brain Injury))) AND ((“Curcumin”[Mesh]) OR (((((((1,6-Heptadiene-3,5-dione, 1,7-bis (4-hydroxy-3-methoxyphenyl)- (E,E)-) OR (Turmeric Yellow)) OR (Yellow, Turmeric)) OR (Curcumin Phytosome)) OR (Phytosome, Curcumin)) OR (Diferuloylmethane)) OR (Mervia)))

### 2.2 Inclusion and exclusion criteria

#### 2.2.1 Inclusion criteria

1. Traumatic Brain Injury (TBI)
2. Animal Experimentation
3. RCTs
4. Administration of Curcumin: Intraperitoneal, Intravenous, or Oral
5. Experimental Design: The experimental group received curcumin, while the control group comprised TBI animals treated with conventional methods or given a blank control treatment.
6. Comprehensive Outcome Measures
7. Outcome Assessment: Continuous numerical variables were used as result indices, with either Standard Mean Difference (SMD) or Mean Difference (MD) employed for data analysis.
8. No Language Restrictions

#### 2.2.2 Exclusion criteria

1. Non-traumatic brain injury
2. Non-Animal Experiments
3. Non-*in vivo* animal studies, *in vitro* studies, human clinical trials, case studies, and experiments lacking control groups.
4. Studies Involving Curcumin Combined with Other Drugs
5. Studies with Incomplete or Unreported Outcome Indicators or Unavailable Full Texts
6. Reviews, systematic reviews, meta-analyses, protocols, conference abstracts, etc.
7. Duplicate Studies Reporting Similar Results from the Same Institution

### 2.3 Study selection

The literature was screened and managed using the EndNote software. Two researchers independently reviewed the literature titles to identify duplicates, review papers, conference papers, protocols, and correspondence. Both researchers assessed the abstracts to determine which literature should be included or excluded. Subsequently, both researchers thoroughly read the remaining literature to make final inclusion decisions. Throughout this process, both researchers independently screened the literature. If both researchers found the same literature, it was included; however, if discrepancies arose, a third researcher intervened to discuss and resolve the matter.

We utilized the EndNote software for literature management, facilitating document screening and exclusion. Two researchers initially assessed the titles for duplication and excluded review papers, conference papers, protocols, and correspondence. Following this, another pair of researchers evaluated the abstracts to determine inclusion or exclusion criteria. Subsequently, the remaining documents were thoroughly reviewed by two researchers to finalize inclusion decisions. Throughout the process, both researchers independently screened the literature. In cases where the literature was identical, it was included; however, if discrepancies arose, a third researcher intervened to discuss and resolve the inclusion status.

### 2.4 Data extraction

Data were recorded using a standardized 11-item data extraction table, specifically selected for inclusion in the study, encompassing the following headings: (1) year of publication (2) country (3) author (4) subject investigated (5) sex (6) mean age (7) mean weight (8) sample size (9) dose or concentration (10) intervention time, and (11) details of inflammatory and oxidation factors. The characteristics of the studies are presented in Table 2.

**Table 2:** Characteristics of the studies included in the meta-analysis.

Serial number	Study	Animal model	Sex	Age (weeks)	Weight	N per group	Dose or concentration	Duration of intervention	Outcome
1	Wu et al. (11)	SD rats *fed* curcumin	M	8	271~301 g	8	500 ppm/d	5 weeks	Protein carbonyl level; BDNF; Synapsin I; CREB
2	Narouiepour et al. (12)	Wistar rats *fed* curcumin	M	N	180~220 g	9/7	50 mg/kg/d	4 weeks	TNF-α; Brain water content
3	Wu et al. (13)	SD rats *fed* curcumin	M	N	200~240 g	6 ~ 8	500 ppm/d	2 weeks	Protein carbonyl level; SOD; Sir2; synapsin I; BDNF; derived from the Cochrane tool, CREB
4	Dai et al. (5)	Adult ICR mice *injected intraperitoneally*	M	6–8	28~32 g	6	100 mg/kg	1 day	SOD; MDA; GPx; mNSS; Bcl-2; Caspase-3; Apoptosis index; Brain water content
5	Sun et al. (14)	SD rats *injected intraperitoneally*	M	8	250~280 g	5	30 mg/kg/d	4 weeks	IL-1β; IL-6; TNF-α; BDNF
6	Laird et al. (15)	CD-1 mice *injected intraperitoneally*	M	8–10	N	6~10	300 mg/kg	1 day	IL-1β; Brain water content
7	Samini et al. (16)	Wistar rats *injected intravenously*	M	N	350~400 g	9	100 mg/kg/d	5 weeks	MDA
8	Zhu et al. (17)	C57BL/6 mice *injected intraperitoneally*	M	8–10	20~25 g	6	100 mg/kg	1 day	IL-1β; IL-6; TNF-α; mNSS; Brain water content;
9	Li et al. (18)	C57BL/6 mice *injected intraperitoneally*	M	8–10	28~32 g	8	200 mg/kg/d	2 weeks	IL-1β; IL-6; TNF-α
10	Sharma et al. (19)	SD rats *fed* curcumin	M	8	N	5~8	500 ppm/d	4 weeks	Sir2
11	Wu et al. (20)	SD rats *fed* curcumin	M	N	200~240 g	5~6	500 ppm/d	4 weeks	4-HNE; BDNF
12	Gao et al. (21)	SD rats *injected intraperitoneally*	M	N	250~280 g	6	50 mg/kg/d	4 weeks	Brain water content; Caspase-3; Apoptosis index; Beclin-1; P62
13	Wei et al. (22)	SD rats *injected intraperitoneally*	M	N	250~280 g	6	50 mg/kg	3 days	SOD; MDA; GPx; Nrf2; Brain water content; Bcl-2; Apoptosis index; Caspase-3
14	Sharma et al. (23, 24)	SD rats *fed* curcumin	M	8	N	5	500 ppm/d	2 weeks	4-HNE
15	Indharty et al. (25)	SD rats *fed* curcumin	N	10–12	280~320 g	11	500 mg/kg/d	1 week	Caspase-3
16	Gao et al. (26)	Adult SD rats *injected intraperitoneally*	M	N	250~300 g	5	100 mg/kg	1 day	MDA; GPx; Bcl-2; Caspase-3; Apoptosis index; Beclin-1
17	Yao-Qi (27)	ICR mice *injected intraperitoneally*	M	8	25~30 g	12	100 mg/kg	2 days	Bcl-2; Caspase-3; P62
18	Dong et al. (28)	C57BL/6 and Nrf2 gene knockout Mice *injected intraperitoneally*	M	8–12	20~26 g	6	50 mg/kg	1 day	IL-1β; IL-6; TNF-α; Brain water content; Bcl-2; Caspase-3; Nrf2; Apoptosis index

SD, Sprague–Dawley; ICR, Institute of Cancer Research; M, Male; N, None; BDNF, Brain-derived neurotrophic factor; CREB, Cyclic AMP-response element*-*binding protein; TNF-α, Tumor necrosis factor-α; Sir2, Silent information regulator 2; SOD, Superoxide dismutase; MDA, Malondialdehyde; GPx, Glutathione peroxidase; mNSS, Modified neurological severity score; Bcl-2, B-cell lymphoma/leukemia 2; IL-1β, Interleukin-1β; IL-6, Interleukin-6; Nrf2, NF-E2-related factor; P62, Autophagy associated protein; 4-HNE, 4-hydroxynonenal.

### 2.5 Risk of bias of individual studies

Two researchers independently assessed the risk of bias using SYRCLE’s risk of bias tool designed for evaluating animal studies. This tool was tailored to address biases specific to animal intervention studies. The assessment considered 10 domains: (1) random sequence generation (2) baseline characteristics (3) allocation concealment (4) random housing (5) blinding of performance bias (6) random outcome assessment (7) blinding of detection bias (8) incomplete outcomes data (9) selective reporting, and (10) bias from other sources. Trials were classified into three levels based on the number of components potentially indicating high risk: high risk (five or more), moderate risk (three or four), and low risk (two or fewer).

### 2.6 Data synthesis

We conducted a meta-analysis using STATA 15.0 software. The analysis utilized a random-effects model, considering the standard mean difference (SMD). To assess the treatment effect on each parameter, we utilized a 95% confidence interval (CI), with significance set at *p* < 0.05. Heterogeneity values were calculated to determine the suitability of included studies for meta-analysis. We quantified heterogeneity using I^2^, considering I^2^ > 50% as substantial if *p* < 0.05. Additionally, a sensitivity analysis was performed to evaluate the impact of individual studies on the 95% CI and 95% CI of SMD.

## 3 Results

### 3.1 Study and identification and selection

**Figure 1:**
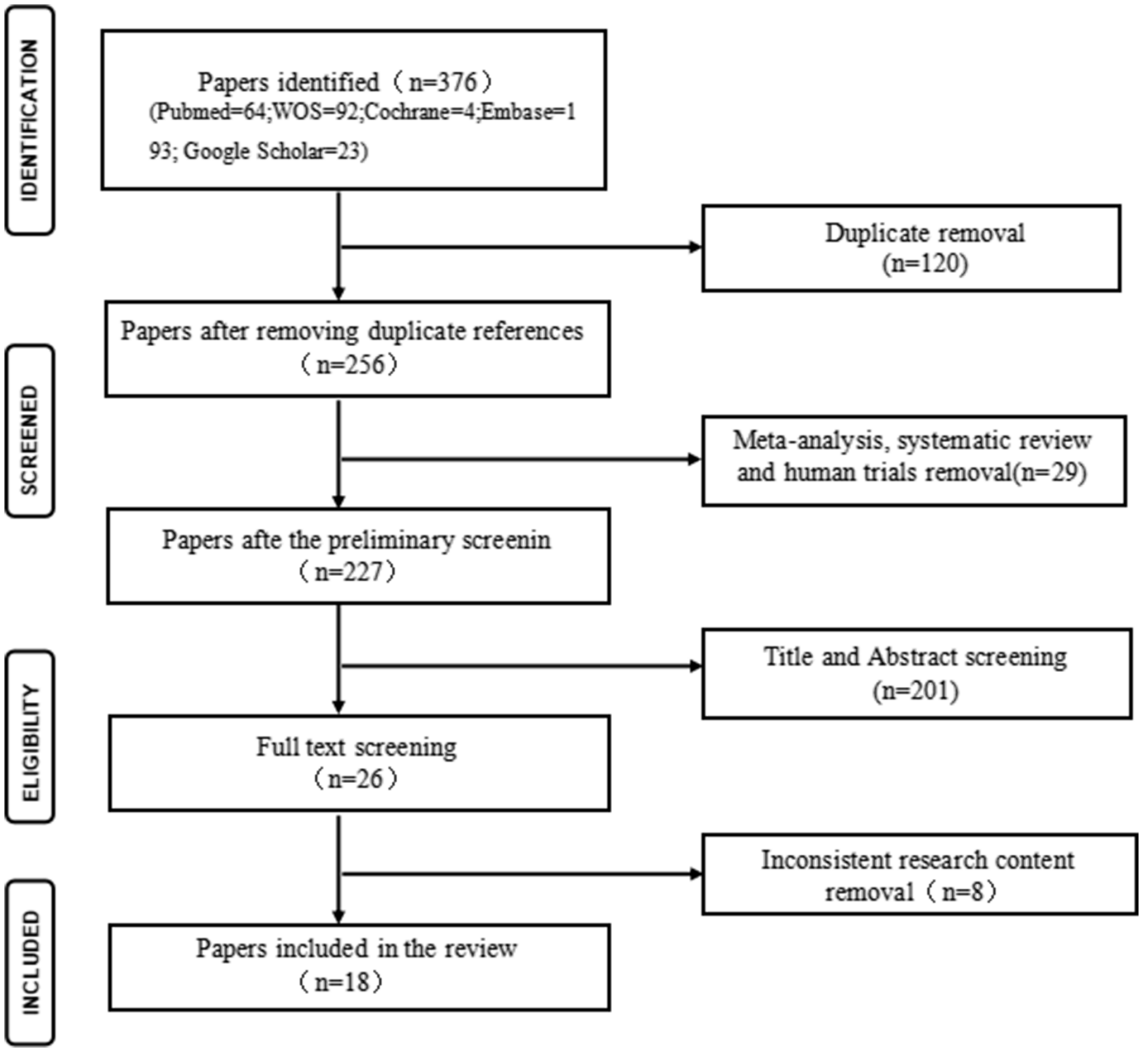
Flow diagram of curcumin study process. WOS: Web of science; n: number.

A total of 376 articles were retrieved from various databases: MEDLINE (*n* = 64), Cochrane (*n* = 4), Embase (*n* = 193), Web of Science (*n* = 92), and Google Scholar (*n* = 23). After eliminating duplicates, 256 unique articles proceeded to the next stage. After screening titles and abstracts, articles including reviews, meta-analyses, human studies, and those not aligned with the research content were excluded, leaving 26 articles for full-text screening. Subsequently, 18 articles meeting the predefined inclusion and exclusion criteria were selected for inclusion in the review. Details of the screening process are presented in Figure 1.

### 3.2 Risk of bias in included studies and publication bias

**Figure 2:**
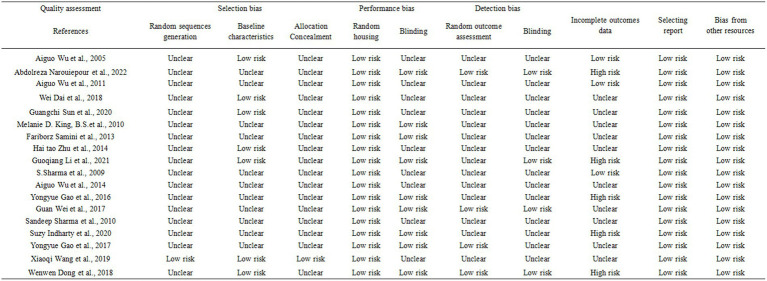
Quality assessment of included animals studies.

We evaluated the risk of bias for the 18 included articles using SYRCLE’s Risk of Bias tool designed for animal studies. Several studies lacked clear descriptions of experiment details, resulting in a classification of “unclear risk of bias.” Across all studies, random sequence generation, allocation concealment, random housing, blinding, random outcome assessment, incomplete outcomes reporting, and selective reporting were incompletely described. However, baseline characteristics and bias from other sources were associated with a low risk of bias. Incomplete outcomes data, primarily due to test animal mortality, were associated with a high risk of bias. Figure 2 depicts the detailed quality assessment (Figure 2). For publication bias, we constructed funnel plots for all outcome measures. No significant publication bias was found by visual inspection (Annex 2: Funnel plot).

### 3.3 Result analysis

#### 3.3.1 Inflammatory factors

##### 3.3.1.1 IL-1β

Five studies (14, 15, 17, 18, 28) investigated the efficacy of curcumin in mitigating the effects of the inflammatory factor IL-1β in TBI. The analysis revealed significant heterogeneity (χ^2^ = 14.33, *p* = 0.006, I^2^ = 72.1%), necessitating the utilization of a random-effects model for statistical analysis. The collective findings demonstrated that curcumin significantly reduced the levels of the inflammatory factor IL-1β in TBI (SMD = −3.22, 95% CI: −4.72, −1.72, *p* = 0.000; Annex 1: Forest map).

The sensitivity analysis for IL-1β indicated that excluding one study at a time and analyzing the standardized mean difference (SMD) from the remaining studies did not significantly alter the efficacy of curcumin in mitigating the inflammatory factor IL-1β in TBI.

##### 3.3.1.2 IL-6

In four studies, the efficacy of curcumin in mitigating the impact of the inflammatory factor IL-6 in TBI was examined (14, 17, 18, 28). Significant heterogeneity was observed (χ^2^ = 25.87, *p* = 0.000, I^2^ = 88.4%), warranting the application of a random effects model for statistical analysis. Meta-analysis results revealed a significant reduction in the inflammatory factor IL-6 associated with TBI following curcumin administration (SMD = −5.31, 95% CI: −8.66 to −1.96, *p* = 0.002; Annex 1: Forest map).

Sensitivity analyses on IL-6 revealed that the exclusion of individual studies and subsequent analysis of the standardized mean difference (SMD) from the remaining studies did not substantially alter the impact of curcumin on reducing the inflammatory factor IL-6 in TBI.

##### 3.3.1.3 TNF-α

In five studies, the efficacy of curcumin in mitigating the impact of the inflammatory factor TNF-α in TBI was examined (12, 14, 17, 18, 28). Significant heterogeneity was observed (χ2 = 11.28, *p* = 0.024, I^2^ = 64.5%), necessitating the application of a random effects model for statistical analysis. The meta-analysis results demonstrated a significant reduction in TNF-α, the inflammatory factor associated with TBI, following curcumin administration (SMD = −2.84, 95% CI: −4.13 to −1.55, *p* = 0.000; Annex 1: Forest map).

Sensitivity analyses on TNF-α revealed that the exclusion of individual studies and subsequent analysis of the standardized mean difference (SMD) from the remaining studies did not significantly alter the impact of curcumin on the inflammatory factor TNF-α in TBI.

#### 3.3.2 Oxidative stress factor

##### 3.3.2.1 SOD

In three studies, we investigated the role of curcumin in augmenting the activity of superoxide dismutase (SOD), a protective factor against oxidative stress, in TBI (5, 13, 22). Significant heterogeneity was observed (χ^2^ = 5.76, *p* = 0.056, I^2^ = 65.3%), necessitating using a random effects model for statistical analysis. The results of the meta-analysis indicated a significant enhancement by curcumin of the oxidative stress factor SOD in TBI (SMD = 4.19, 95% CI: 2.30 to 6.07, *p* = 0.000; Annex 1: Forest map).

Sensitivity analyses on SOD revealed that the exclusion of individual studies and subsequent analysis of the standardized mean difference (SMD) from the remaining studies did not significantly alter the effect of curcumin on enhancing the oxidative stress factor SOD in TBI.

##### 3.3.2.2 Sir2

In two studies, we investigated how curcumin enhances the effects of the oxidative stress protective factor Sir2 in TBI (13, 19). Statistical analysis using the random effects model was conducted due to the observed heterogeneity (χ^2^ = 1.63, *p* = 0.201, I^2^ = 38.8%). The meta-analysis results indicated a significant enhancement by curcumin of the oxidative stress factor Sir2 in TBI (SMD = 5.15, 95% CI: 3.16 to 7.15, *p* = 0.000; Annex 1: Forest map).

Sensitivity analysis of Sir2 demonstrated that excluding individual studies and subsequent analysis of the standardized mean difference (SMD) from the remaining studies did not significantly alter the impact of curcumin on enhancing the oxidative stress factor Sir2 in TBI.

##### 3.3.2.3 GPx

In three studies, we investigated how curcumin enhances the effects of the oxidative stress protective factor GPx in TBI (5, 22, 26). Given the absence of heterogeneity (χ^2^ = 1.78, *p* = 0.410, I^2^ = 0.0%), statistical analysis was conducted using the random effects model. The meta-analysis results indicated a significant enhancement by curcumin of the effect of the oxidative stress protective factor GPx on TBI (SMD = 4.13, 95% CI: 3.05 to 5.22, *p* = 0.000; Annex 1: Forest map).

Sensitivity analysis of GPx revealed that omitting one study at a time and analyzing the standardized mean difference (SMD) from the remaining studies did not significantly affect the enhancement of TBI oxidative stress protective factor GPx by curcumin.

##### 3.3.2.4 Nrf2

In two studies, we investigated how curcumin enhances the effect of Nrf2, a protective factor against oxidative stress in TBI (22, 28). Given the minimal heterogeneity (χ2 = 1.12, *p* = 0.290, I2 = 10.6%), statistical analysis was conducted using the random effects model. The meta-analysis results revealed a significant enhancement by curcumin of the effect of Nrf2 on oxidative stress in TBI (SMD = 2.92, 95% CI: 1.85 to 3.99, *p* = 0.000; Annex 1: Forest map).

Sensitivity analysis on Nrf2 demonstrated that excluding individual studies and subsequent analysis of the standardized mean difference (SMD) from the remaining studies did not significantly alter the enhancement by curcumin of Nrf2, a protective factor against oxidative stress in TBI.

##### 3.3.2.5 MDA

In four studies, the efficacy of curcumin in mitigating the effects of the oxidative stress damage factor MDA in TBI was examined (5, 16, 22, 26). With no observed heterogeneity (χ^2^ = 2.31, *p* = 0.510, I^2^ = 0.0%), statistical analysis was conducted using the random effects model. The meta-analysis results demonstrated a significant reduction by curcumin in the effect of TBI oxidative stress factor MDA (SMD = −2.92, 95% CI: −3.66 to −2.18, *p* = 0.000; Annex 1: Forest map).

Sensitivity analysis on MDA indicated that the exclusion of individual studies and subsequent analysis of the standardized mean difference (SMD) from the remaining studies did not significantly affect the impact of curcumin on reducing the oxidative stress damage factor MDA in TBI.

##### 3.3.2.6 4-HNE

In two studies, the impact of curcumin on the oxidative stress damage factor 4-HNE in TBI was examined (20, 23, 24). The random effects model was employed for statistical analysis due to the mild heterogeneity observed (χ^2^ = 2.39, *p* = 0.122, I^2^ = 58.2%). The meta-analysis results indicated a significant reduction by curcumin in the effect of oxidative stress damage factor 4-HNE on TBI (SMD = −9.21, 95% CI: −14.58 to −3.84, *p* = 0.001; Annex 1: Forest map).

Sensitivity analysis on 4-HNE demonstrated that excluding individual studies and subsequent analysis of the standardized mean difference (SMD) from the remaining studies did not significantly affect the impact of curcumin on reducing the oxidative stress damage factor 4-HNE in TBI.

##### 3.3.2.7 Protein carbonyl level

In two studies, the efficacy of curcumin in reducing oxidative protein levels in TBI was examined (11, 13). Significant heterogeneity was observed (χ^2^ = 8.22, *p* = 0.004, I^2^ = 87.8%), warranting using the random effects model for statistical analysis. The meta-analysis results revealed a significant decrease by curcumin in the level of oxidized protein in TBI (SMD = −9.83, 95% CI: −18.36 to −1.30, *p* = 0.024; Annex 1: Forest map).

Sensitivity analysis of oxidized protein levels demonstrated that excluding individual studies and subsequent analysis of the standardized mean difference (SMD) from the remaining studies did not significantly impact curcumin’s ability to reduce oxidized protein levels in TBI.

#### 3.3.3 Neuroprotective factor

##### 3.3.3.1 Brain water content

In seven studies, the impact of curcumin on reducing brain edema after TBI was investigated (5, 12, 15, 17, 21, 22, 28). Significant heterogeneity was observed (χ^2^ = 22.11, *p* = 0.001, I^2^ = 72.9%), leading to the utilization of the random effects model for statistical analysis. Meta-analysis results revealed a significant reduction by curcumin in the effect of TBI cerebral edema (SMD = −4.17, 95% CI: −5.53 to −2.82, *p* = 0.000; Annex 1: Forest map).

Sensitivity analysis of brain edema showed that the exclusion of individual studies and subsequent analysis of the standardized mean difference (SMD) from the remaining studies did not significantly impact the effect of curcumin on reducing brain edema after TBI.

##### 3.3.3.2 mNSS

In two studies, the impact of curcumin on modified neurological severity score (mNSS) in TBI was assessed (5, 17). Significant heterogeneity was observed (χ^2^ = 8.25, *p* = 0.004, I^2^ = 87.9%), prompting the use of the random effects model for statistical analysis. The meta-analysis results suggested a potential effect of curcumin on TBI mNSS (SMD = −3.08, 95% CI: −7.20 to −1.05, *p* = 0.144); however, further analysis with an increased sample size is needed to confirm this effect (Annex 1: Forest map).

Sensitivity analysis of mNSS indicated that excluding the study by Zhu et al. (17) resulted in a reduction of TBI mNSS with curcumin while excluding the study by Dai et al. yielded inconclusive findings regarding the effect of curcumin on TBI mNSS. Given the limited sample size, additional research with a larger sample is necessary for a conclusive discussion.

##### 3.3.3.3 Synapsin I

In two studies, the impact of curcumin on enhancing the role of synaptophysin 1 in TBI was investigated (11, 13). Significant heterogeneity was observed (χ^2^ = 5.00, *p* = 0.025, I^2^ = 80%), leading to using the random effects model for statistical analysis. The meta-analysis results indicated a significant enhancement by curcumin in the effect of synaptophysin 1 in TBI (SMD = 2.98, 95% CI: 0.49 to 5.47, *p* = 0.019; Annex 1: Forest map).

Proportional sensitivity analysis of neuron survival demonstrated that omitting one study at a time and analyzing the standardized mean difference (SMD) from the remaining studies did not significantly alter the effect of curcumin on enhancing synaptophysin 1 in TBI.

##### 3.3.3.4 BDNF

In four studies, the efficacy of curcumin in enhancing the effects of BDNF in TBI was investigated (11, 13, 14, 20). Significant heterogeneity was observed (χ^2^ = 9.28, *p* = 0.026, I^2^ = 67.7%), leading to the utilization of the random effects model for statistical analysis. The meta-analysis results demonstrated that curcumin significantly augmented the effect of BDNF on TBI (SMD = 3.99, 95% CI: 2.19 to 5.79, *p* = 0.000; Annex 1: Forest map).

Sensitivity analyses of BDNF showed that omitting one study at a time and analyzing the standardized mean difference (SMD) from the remaining studies did not significantly alter the effect of curcumin in enhancing BDNF in TBI.

##### 3.3.3.5 CREB

In two studies, the impact of curcumin on enhancing the effects of CREB in TBI was examined (11, 13). Minimal heterogeneity was observed (χ^2^ = 1.02, *p* = 0.312, I^2^ = 2.0%). Thus, the fixed-effect model was employed for statistical analysis. The meta-analysis results revealed that curcumin significantly augmented the effect of CREB in TBI (SMD = 2.81, 95% CI: 1.78 to 3.84, *p* = 0.000).

Sensitivity analysis of CREB indicated that excluding one study at a time and analyzing the standardized mean difference (SMD) from the remaining studies did not substantially alter the effect of curcumin on enhancing CREB in TBI (Annex 1: Forest map).

#### 3.3.4 Autophagy and apoptosis factors

##### 3.3.4.1 Bcl-2

In five studies, we investigated how curcumin enhances the effects of Bcl-2, an apoptosis factor in TBI (5, 22, 26–28). Significant heterogeneity was observed (χ^2^ = 13.23, *p* = 0.010, I^2^ = 69.8%), necessitating the utilization of the random effects model for statistical analysis. The meta-analysis results revealed that curcumin significantly augmented the effect of the TBI apoptosis factor Bcl-2 (SMD = 2.98, 95% CI: 1.69 to 4.27, *p* = 0.000; Annex 1: Forest map).

Sensitivity analysis for GPx demonstrated that excluding one study at a time and analyzing the standardized mean difference (SMD) from the remaining studies did not substantially affect curcumin’s enhancement of the TBI apoptosis factor Bcl-2.

##### 3.3.4.2 Beclin-1

In two studies, the efficacy of curcumin in enhancing the effect of Beclin-1 in TBI was investigated (21, 26). No significant heterogeneity was observed (χ^2^ = 0.04, *p* = 0.84, I^2^ = 0.0%), warranting using the random effects model for statistical analysis. The meta-analysis showed that curcumin significantly augmented the Beclin-1 effect in TBI (SMD = 6.08, 95% CI: 3.92 to 8.23, *p* = 0.000; Annex 1: Forest map).

Sensitivity analysis for Beclin-1 indicated that omitting one study at a time and analyzing the standardized mean difference (SMD) from the remaining studies did not substantially affect curcumin’s enhanced Beclin-1 effect in TBI.

##### 3.3.4.3 Caspase-3

In seven studies, we examined the impact of curcumin on reducing the activity of the apoptosis factor caspase-3 in TBI (5, 21, 22, 25–28). Significant heterogeneity was observed (χ2 = 17.11, *p* = 0.009, I2 = 64.9%), leading to the adoption of the random effects model for statistical analysis. The meta-analysis showed that curcumin significantly reduced the apoptosis of TBS cells by targeting the caspase-3 pathway (SMD = −4.84, 95% CI: −6.18 to −3.50, *p* = 0.000; Annex 1: Forest map).

Sensitivity analysis for caspase-3 demonstrated that excluding one study at a time and subsequent analysis of the standardized mean difference (SMD) from the remaining studies did not significantly impact curcumin’s efficacy in reducing caspase-3 activity in TBI cells.

##### 3.3.4.4 Apoptosis index

In five studies, we investigated the impact of curcumin on reducing the apoptosis index of cerebral cortex cells following TBI (5, 21, 22, 26, 28). Significant heterogeneity was observed (χ^2^ = 22.48, *p* = 0.000, I^2^ = 82.2%), prompting the use of the random effects model for statistical analysis. The meta-analysis results demonstrated a significant reduction in the apoptosis index of cerebral cortex cells following TBI with curcumin treatment (SMD = −4.80, 95% CI: −7.11 to −2.48, p = 0.000; Annex 1: Forest map).

Sensitivity analysis of the cortical apoptotic index indicated that excluding one study at a time and analyzing the standardized mean difference (SMD) from the remaining studies did not significantly alter the observed effect of curcumin on reducing the cortical apoptotic index after TBI.

##### 3.3.4.5 P62

In two studies, we investigated how curcumin mitigates the impact of the apoptosis factor P62 in TBI (21, 27). Significant heterogeneity was observed (χ^2^ = 3.12, *p* = 0.077, I^2^ = 67.9%), necessitating the utilization of the random effects model for statistical analysis. The meta-analysis results revealed a significant reduction in the effect of TBI apoptosis factor P62 with curcumin treatment (SMD = −5.30, 95% CI: −8.63, −1.97, *p* = 0.002; Annex 1: Forest map).

Sensitivity analysis for P62 showed that excluding one study at a time and analyzing the standardized mean difference (SMD) from the remaining studies did not significantly alter the effect of curcumin in reducing the impact of TBI apoptosis factor P62. The meta-analysis results are presented in Table 3.

**Table 3:** Meta-analysis results of curcumin in the treatment of inflammation and oxidative stress in traumatic brain injury.

Index		Number of references	Number of experimental group	Number of control group	Heterogeneity test result		Effect model	Meta-analysis result	
					I^2^	*P*		SMD (95%CI)	*P*
Inflammatory factors	IL-1β	5	31~35	31~35	72.1%	0.006	Random	−3.22 (−4.72, −1.72)	0.000*
	IL-6	4	25	25	88.4%	0.000	Random	−5.31 (−8.66, −1.96)	0.002*
	TNF-α	5	34	32	64.5%	0.024	Random	−2.84 (−4.13, −1.55)	0.000*
Oxidative stress factor	SOD	3	18~20	18~20	65.3%	0.056	Random	4.19 (2.30, 6.07)	0.000*
	Sir2	2	11~16	11~16	38.8%	0.201	Random	5.15 (3.16, 7.15)	0.000*
	GPx	3	17	17	0.0%	0.410	Random	4.13 (3.05, 5.22)	0.000*
	Nrf2	2	12	12	10.6%	0.290	Random	2.92 (1.85, 3.99)	0.000*
	MDA	4	26	26	0.0%	0.510	Random	−2.92 (−3.66, −2.18)	0.000*
	4-HNE	2	10~11	10~11	58.2%	0.122	Random	−9.21 (−14.5, −3.84)	0.001*
	Protein carbonyl level	2	14~16	14~16	87.8%	0.004	Random	−9.83 (−18.36, −1.30)	0.024*
Neuroprotective factor	Brain water content	7	45~49	43~47	72.9%	0.001	Random	−4.17 (−5.53, −2.82)	0.000*
	mNSS	2	12	12	87.9%	0.004	Random	−3.08 (−7.20, −1.05)	0.144
	Synapsin I	2	14~16	14~16	80%	0.025	Random	2.98 (0.49, 5.47)	0.019*
	BDNF	4	24~27	24~27	67.7%	0.026	Random	3.99 (2.19, 5.79)	0.000*
	CREB	2	14~16	14~16	2.0%	0.312	Random	2.81 (1.78, 3.84)	0.000*
Autophagy and apoptosis factors	Bcl-2	5	35	35	69.8%	0.010	Random	2.98 (1.69, 4.27)	0.000*
	Beclin-1	2	11	11	0.0%	0.840	Random	6.08 (3.92, 8.23)	0.000*
	Caspase-3	7	52	52	64.9%	0.009	Random	−4.84 (−6.18, −3.50)	0.000*
	Apoptosis index	5	29	29	82.2%	0.000	Random	−4.80 (−7.11, −2.48)	0.000*
	P62	2	18	18	67.9%	0.077	Random	−5.30 (−8.63, −1.97)	0.002*

SMD, Standardized mean difference; CI, Confidence interval; BDNF, Brain-derived neurotrophic factor; CREB, Cyclic AMP-response element-binding protein; TNF-α, Tumor necrosis factor-α; Sir2, Silent information regulator 2; SOD, Superoxide dismutase; MDA, Malondialdehyde; GPx, Glutathione peroxidase; mNSS, Modified neurological severity score; Bcl-2, B-cell lymphoma/leukemia 2; IL-1β, Interleukin-1β; IL-6, Interleukin-6; Nrf2, NF-E2-related factor; P62, Autophagy associated protein; 4-HNE, 4-hydroxynonenal. ^*^P < 0.05, the difference was statistically significant. * Indicates that *p* < 0.05, the difference was statistically significant.

## 4 Discussion

This review systematically examined the impact of curcumin on inflammation and oxidative stress responses induced by TBI across various animal models, encompassing 18 animal studies involving 64 mice and 194 rats. Our findings indicate that curcumin significantly mitigates the effects of inflammatory cytokines such as IL-1β, IL-6, and TNF-α. Moreover, curcumin enhances the efficacy of oxidative stress factors, including SOD, Sir2, GPx, and Nrf2. Conversely, it reduces the effects of oxidative stress factors MDA, 4-HNE, and protein carbonyl levels. Regarding neurological function, curcumin reduces brain edema, increases neuron survival rates, and augmentation of the effects of synapsin I, BDNF, and CREB, but has no effect in reducing mNSS. Due to the limited literature involved, more high-quality studies are needed to further verify whether curcumin can reduce neural function scores. Furthermore, curcumin enhances the effects of Beclin-1 and Bcl-2 in autophagy and apoptosis, respectively, while diminishing the effects of caspase-3, apoptosis index, and P62.

In the study of TBI animal experimental models, curcumin, administered in various concentrations, durations, and routes, significantly reduces pro-inflammatory cytokines in the experimental group, improves nerve function following TBI, and mitigates the inflammatory response. Bassani et al. (29) demonstrated that curcumin effectively reduces neuroinflammation in models of neurodegenerative and neuroinflammatory diseases. Additional studies (15, 17, 30) conducted in animal experimental models have shown that curcumin inhibits the TLR4/NF-κB signaling pathway, thereby down-regulating inflammatory cytokines such as IL-1β, IL-6, and TNF-α, consequently reducing inflammatory damage. Narouiepour et al. (12), Sun et al. (14), and Li et al. (18) suggested that curcumin regulates transcription factors such as STAT, NF-jB, and AP1, activates ERK1/2 and p38 signaling pathways, and suppresses the expression of pro-inflammatory cytokines IL-1β, IL-6, and TNF-α by inhibiting the TLR4-MAPK/NF-jB pathways, thereby mitigating chronic inflammation following TBI. Additionally, Dong et al. (28) and Wu et al. (31) found that curcumin activates the Nrf2 signaling pathway, alleviating the expression of inflammatory response factors in TBI, reducing inflammatory mediators, and exerting a neuroprotective effect.

The results of this meta-analysis confirm that curcumin effectively reduces oxidative stress levels following TBI. Curcumin achieves this through four main mechanisms: (1) reducing the levels of malondialdehyde (MDA), 4-hydroxynonenal (4-HNE), and protein carbonyls, which are significant products of lipid peroxidation (11, 32); (2) improving the activity of antioxidant enzymes glutathione peroxidase (GPx) and superoxide dismutase (SOD); (3) enhancing the level of nuclear factor erythroid 2-related factor 2 (Nrf2), a key regulator of endogenous antioxidant stress; and (4) promoting the level of sirtuin 2 (Sir2), an essential regulator of genomic stability and cell homeostasis. Curcumin, a widely used antioxidant with neuroprotective properties (21, 23, 24), has been shown to inhibit lipid peroxide formation in the presence of lipid peroxidation-inducing drugs. Moreover, curcumin reduces levels of MDA, 4-HNE, and protein carbonyls, restores mitochondrial oxidation function, stabilizes cell membrane homeostasis, and thereby mitigates the oxidative stress response following TBI (16, 20). Gao et al. (21) demonstrated that curcumin treatment significantly reduces MDA levels and induces GPx activation, thereby ameliorating TBI-induced oxidative stress in rat models. Studies provide evidence that curcumin is a potent activator of Nrf2 both *in vivo*; (33) and *in vitro* (34), enhancing Nrf2 activation in the brain (35, 36). Xie et al. (37) found that curcumin activates Nrf2 to translocate to the nucleus and upregulate downstream enzymes, protecting rats from acute liver injury induced by lipopolysaccharide/D-galactosamine. Dong et al. injected curcumin into the abdominal cavity of a mouse wound model, observing improved Nrf2 expression and transport, along with upregulated downstream antioxidant enzymes, exerting a protective effect on nerves (5, 19, 28). Sir2, an NAD+-dependent deacetylase, participates in transcription factors governing energy homeostasis, oxidative stress response, metabolism, and gene expression to maintain normal brain function (38). Curcumin counteracts TBI-induced reductions in Sir2 levels and markers of energy metabolism, thus ameliorating TBI-induced oxidative stress (13, 19).

The results of this meta-analysis indicate that curcumin enhances the levels of the neurotrophic factor BDNF, promotes the upregulation of BDNF downstream proteins synaptophysin I and CREB, partially reduces brain edema, and mitigates the mNSS. Narouiepour et al. (12) demonstrated that combining curcumin with neural stem cell therapy significantly mitigates TBI-induced brain edema and reduces reactive astrocyte numbers. Laird et al. (15) found that administering curcumin alleviates brain edema, reduces the expression of the glial water channel AQP4 (which promotes brain edema), and improves neurological prognosis post-injury. Sharma et al. (23, 24) reported that curcumin reduces brain edema post-injury and promotes cell membrane and energy homeostasis, consequently impacting synaptic plasticity. Zhu et al. (17) showed that curcumin alleviates acute inflammatory injury, reduces brain edema, and significantly decreases mNSS and neuronal mortality by inhibiting the TLR4/MyD88/NF-κB signaling pathway in experimental TBI. Studies have highlighted BDNF’s efficacy in reducing inflammation and increasing hippocampal neurogenesis through the tropomyosin receptor kinase B (TrkB) signaling pathway (20, 39, 40). Curcumin activates the antioxidant and anti-inflammatory properties of the BDNF/TrkB-dependent pathway, modulates BDNF/TrkB signaling, promotes nerve regeneration in the hippocampus, and significantly reverses 6-hydroxydopamine-induced hippocampal neuron damage (41, 42). Furthermore, curcumin promotes the phosphorylation of the BDNF receptor TrkB in the hippocampus, thereby enhancing the effect of DHA on TBI (43). Combining curcumin and DHA mitigates TBI-related learning disabilities via the BDNF pathway. Dietary supplementation with curcumin may protect against cognitive impairment after TBI by upregulating BDNF-related molecules such as synaptophysin I and CREB (43–45).

Curcumin enhances the expression of autophagy-associated markers such as P62 and Beclin-1, reduces the level of the pro-apoptotic protein caspase-3, promotes the anti-apoptotic protein Bcl-2, and diminishes the apoptotic index of the cerebral cortex. Autophagy is a precise regulatory process involving the degradation and recycling of damaged organelles and cytoplasmic substances, widely present in eukaryotic cells (46–48). P62 indicates autophagy flux, while Beclin-1 denotes autophagosome formation (21, 49). Damaged mitochondria can undergo autophagic degradation after TBI, reducing oxidative stress burden (50). The present study reveals the activation of autophagy pathways in rats treated with tetrahydro curcumin following TBI, achieved by upregulating Beclin-1 expression and downregulating P62 expression (21). Curcumin can penetrate the blood–brain barrier after peripheral injection, modulate autophagy, and exhibit potent antioxidant properties (51). Gao Yongyue et al. discovered that intraperitoneal injection of curcumin 30 min after TBI increased the levels of the autophagy-related protein Beclin-1, activated autophagy, elevated mitochondrial anti-apoptotic protein Bcl-2 levels, reduced caspase-3 content, and protected the brain from mitochondrial apoptosis, with the most significant effect observed 24 h after TBI (15, 27, 28).

This meta-analysis (27, 28) demonstrated that curcumin enhances Bcl-2 levels and reduces active Caspase-3 levels after TBI, mitigating apoptosis around cortical contusions (22), and thereby inhibiting neuronal apoptotic activity in the injured cerebral cortex. Apoptosis, a classical mode of programmed cell death, is a gene-regulated, energy-dependent, and orderly process that can be categorized into Caspase-dependent and non-Caspase-dependent pathways based on Caspase involvement. The Caspase-dependent pathway is the primary mode of apoptosis (25). Caspase-3, also known as the executioner of apoptosis (52), plays a pivotal role in this process. Studies have revealed the PI3K/AKT signaling pathway as one of the anti-apoptotic mechanisms after TBI (53), primarily characterized by the inhibition of the anti-apoptotic protein Bcl-2 and significant activation of the Caspase-3 protein (54, 55). Guan Wei et al. and Gao Yongyue et al. found that tetrahydro curcumin activates the PI3K/AKT pathway, exerting anti-apoptotic effects after TBI (5, 21, 22). Furthermore, studies have indicated that curcumin can reduce neuronal apoptosis in patients with human immunodeficiency virus type 1 (HIV-1) by promoting the expression of heat shock protein 70 (HSP 70) (25, 56).

## 5 Advantages and limitations

### 5.1 Advantages

Curcumin, a neuroprotective agent, is relatively underexplored in TBI research, making our topic selection novel. Our meta-analysis was conducted on existing animal experiments with multiple participants, employing rigorous data extraction and screening methods, rendering the results relatively reliable. This study holds particular value in guiding clinical human experiments.

### 5.2 Limitations

Our study shares some common limitations with the studies it incorporates. Despite our efforts to control for heterogeneity among the included original studies, variation between studies is inevitable, including factors such as animal species, age, sex, weight, modeling techniques, and curcumin treatment routes. Additionally, our study’s limited literature and sample size may introduce errors in the results.

## 6 Conclusion

In our study, curcumin exhibits potent anti-inflammatory, antioxidant, and anti-apoptotic effects, suggesting its potential for treating TBI in humans. However, future clinical trials are needed to evaluate its safety and efficacy in detail. Due to the limitation of the quality and quantity of the included literature, the conclusion of this study still needs to be confirmed by high-quality clinical studies with large samples.

## Data availability statement

The original contributions presented in the study are included in the article/Supplementary material, further inquiries can be directed to the corresponding author.

## Author contributions

JG: Data curation, Methodology, Supervision, Conceptualization, Formal analysis, Project administration, Validation, Investigation, Funding acquisition, Resources, Visualization, Writing – original draft, Writing – review & editing. ZL: Data curation, Methodology, Supervision, Conceptualization, Formal analysis, Writing – original draft. YY: Data curation, Methodology, Conceptualization, Formal analysis, Investigation, Writing – original draft. LF: Data curation, Methodology, Conceptualization, Formal analysis, Investigation, Writing – original draft. MY: Data curation, Methodology, Conceptualization, Formal analysis, Investigation, Writing – original draft. ZW: Data curation, Methodology, Conceptualization, Formal analysis, Investigation, Writing – original draft.

## References

[1] Stocchetti N . Traumatic brain injury: problems and opportunities. Lancet Neurol. (2014) 13:14–6. doi: 10.1016/S1474-4422(13)70280-1 24331787

[2] Xiong Y Mahmood A Chopp M. Animal models of traumatic brain injury. Nat Rev Neurosci. (2013) 14:128–42. doi: 10.1038/nrn3407, PMID: 23329160 PMC3951995

[3] Angeloni C Prata C Dalla SF Piperno R Hrelia S. Traumatic brain injury and NADPH oxidase: a deep relationship. Oxidative Med Cell Longev. (2015) 2015:370312. doi: 10.1155/2015/370312, PMID: 25918580 PMC4397034

[4] Galgano M Toshkezi G Qiu X Russell T Chin L Zhao LR. Traumatic brain injury: current treatment strategies and future endeavors. Cell Transplant. (2017) 26:1118–30. doi: 10.1177/0963689717714102, PMID: 28933211 PMC5657730

[5] Dai W Wang H Fang J Zhu Y Zhou J Wang X . Curcumin provides neuroprotection in model of traumatic brain injury via the Nrf2-ARE signaling pathway. Brain Res Bull. (2018) 140:65–71. doi: 10.1016/j.brainresbull.2018.03.020, PMID: 29626606

[6] Forouzanfar F Read MI Barreto GE Sahebkar A. Neuroprotective effects of curcumin through autophagy modulation. IUBMB Life. (2020) 72:652–64. doi: 10.1002/iub.2209, PMID: 31804772

[7] Ullah F Liang A Rangel A Gyengesi E Niedermayer G Münch G. High bioavailability curcumin: an anti-inflammatory and neurosupportive bioactive nutrient for neurodegenerative diseases characterized by chronic neuroinflammation. Arch Toxicol. (2017) 91:1623–34. doi: 10.1007/s00204-017-1939-4, PMID: 28204864

[8] Razavi BM Ghasemzadeh RM Hosseinzadeh H. A review of therapeutic potentials of turmeric (*Curcuma longa*) and its active constituent, curcumin, on inflammatory disorders, pain, and their related patents. Phytother Res. (2021) 35:6489–513. doi: 10.1002/ptr.7224 34312922

[9] Zahedi H Hosseinzadeh Attar MJ Shadnoush M Sahebkar A Barkhidarian B Sadeghi O . Effects of curcuminoids on inflammatory and oxidative stress biomarkers and clinical outcomes in critically ill patients: a randomizeddouble-blind placebo-controlled trial. Phytother Res. (2021) 35:4605–15. doi: 10.1002/ptr.7179, PMID: 34080237

[10] Eghbaliferiz S Farhadi F Barreto GE Majeed M Sahebkar A. Effects of curcumin on neurological diseases: focus on astrocytes. Pharmacol Rep. (2020) 72:769–82. doi: 10.1007/s43440-020-00112-3, PMID: 32458309

[11] Wu A Ying Z Gomez-Pinilla F. Dietary curcumin counteracts the outcome of traumatic brain injury on oxidative stress, synaptic plasticity, and cognition. Exp Neurol. (2006) 197:309–17. doi: 10.1016/j.expneurol.2005.09.004, PMID: 16364299

[12] Narouiepour A Ebrahimzadeh-Bideskan A Rajabzadeh G Gorji A Negah SS. Neural stem cell therapy in conjunction with curcumin loaded in niosomal nanoparticles enhanced recovery from traumatic brain injury. Sci Rep. (2022) 12:3572. doi: 10.1038/s41598-022-07367-1, PMID: 35246564 PMC8897489

[13] Wu A Ying Z Schubert D Gomez-Pinilla F. Brain and spinal cord interaction. Neurorehabil Neural Repair. (2011a) 25:332–42. doi: 10.1177/1545968310397706, PMID: 21343524 PMC3258099

[14] Sun G Miao Z Ye Y Zhao P Fan L Bao Z . Curcumin alleviates neuroinflammation, enhances hippocampal neurogenesis, and improves spatial memory after traumatic brain injury. Brain Res Bull. (2020) 162:84–93. doi: 10.1016/j.brainresbull.2020.05.009, PMID: 32502596

[15] Laird MD Sukumari-Ramesh S Swift AE Meiler SE Vender JR Dhandapani KM. Curcumin attenuates cerebral edema following traumatic brain injury in mice: a possible role for aquaporin-4? J Neurochem. (2010) 113:637–48. doi: 10.1111/j.1471-4159.2010.06630.x, PMID: 20132469 PMC2911034

[16] Samini F Samarghandian S Borji A Mohammadi G Bakaian M. Curcumin pretreatment attenuates brain lesion size and improves neurological function following traumatic brain injury in the rat. Pharmacol Biochem Behav. (2013) 110:238–44. doi: 10.1016/j.pbb.2013.07.019, PMID: 23932920

[17] Zhu HT Bian C Yuan JC Chu WH Xiang X Chen F . Curcumin attenuates acute inflammatory injury by inhibiting the TLR4/MyD88/NF-kappaB signaling pathway in experimental traumatic brain injury. J Neuroinflammation. (2014) 11:59. doi: 10.1186/1742-2094-11-59, PMID: 24669820 PMC3986937

[18] Li G Duan L Yang F Yang L Deng Y Yu Y . Curcumin suppress inflammatory response in traumatic brain injury via p38/MAPK signaling pathway. Phytother Res. (2022) 36:1326–37. doi: 10.1002/ptr.7391 35080289

[19] Sharma S Zhuang Y Ying Z Wu A Gomez-Pinilla F. Dietary curcumin supplementation counteracts reduction in levels of molecules involved in energy homeostasis after brain trauma. Neuroscience. (2009) 161:1037–44. doi: 10.1016/j.neuroscience.2009.04.042, PMID: 19393301 PMC2805661

[20] Wu A Ying Z Gomez-Pinilla F. Dietary strategy to repair plasma membrane after brain trauma. Neurorehabil Neural Repair. (2014) 28:75–84. doi: 10.1177/1545968313498650, PMID: 23911971

[21] Gao YM Li JMP Wu LMP Zhou CMP Wang QMP Li XM . Tetrahydrocurcumin provides neuroprotection in rats after traumatic brain injury: autophagy and the PI3K/AKT pathways as a potential mechanism. J Dermatol Surg. (2016) 206:67–76. doi: 10.1016/j.jss.2016.07.014, PMID: 27916377

[22] Wei G Chen B Lin Q Li Y Luo L He H . Tetrahydrocurcumin provides neuroprotection in experimental traumatic brain injury and the Nrf2 signaling pathway as a potential mechanism. Neuroimmunomodulation. (2017) 24:348–55. doi: 10.1159/000487998, PMID: 29669346

[23] Sharma S Ying Z Gomez-Pinilla F. A pyrazole curcumin derivative restores membrane homeostasis disrupted after brain trauma. Exp Neurol. (2010) 226:191–9. doi: 10.1016/j.expneurol.2010.08.027, PMID: 20816821 PMC3225197

[24] Sharma V Nehru B Munshi A Jyothy A. Antioxidant potential of curcumin against oxidative insult induced by pentylenetetrazol in epileptic rats. Methods Find Exp Clin Pharmacol. (2010) 32:227–32. doi: 10.1358/mf.2010.32.4.1452090, PMID: 20508869

[25] Indharty S Japardi I Tandean S AMP S Loe ML. Efficacy of neuroprotection from curcumin through heat shock protein 70 induction in traumatic brain injury-rat model. Macedonian J Med Sci. (2020) 8:593–6. doi: 10.3889/oamjms.2020.4933

[26] Gao Y Zhuang Z Gao S Li X Zhang Z Ye Z . Tetrahydrocurcumin reduces oxidative stress-induced apoptosis via the mitochondrial apoptotic pathway by modulating autophagy in rats after traumatic brain injury. Am J Transl Res. (2017) 9:887–99. PMID: 28386319 PMC5375984

[27] Yao-Qi W . Effect of curcumin on autophagy apoptosis and histocytic renovation in mice after traumatic brain injury. Chin J Contemp Neurol Neurosurg. (2019) 19:581–587. doi: 10.3969/j.issn.1672-6731.2019.08.008

[28] Dong W Yang B Wang L Li B Guo X Zhang M . Curcumin plays neuroprotective roles against traumatic brain injury partly via Nrf2 signaling. Toxicol Appl Pharmacol. (2018) 346:28–36. doi: 10.1016/j.taap.2018.03.020, PMID: 29571711

[29] Bassani TB Turnes JM Moura ELR Bonato JM Cóppola-Segovia V Zanata SM . Effects of curcumin on short-term spatial and recognition memory, adult neurogenesis and neuroinflammation in a streptozotocin-induced rat model of dementia of Alzheimer’s type. Behav Brain Res. (2017) 335:41–54. doi: 10.1016/j.bbr.2017.08.014, PMID: 28801114

[30] Fan L Dong J He X Zhang C Zhang T. Bone marrow mesenchymal stem cells-derived exosomes reduce apoptosis and inflammatory response during spinal cord injury by inhibiting the TLR4/MyD88/NF-κB signaling pathway. Hum Exp Toxicol. (2021) 40:1612–23. doi: 10.1177/09603271211003311, PMID: 33779331

[31] Wu A Ying Z Gomez-Pinilla F. Dietary omega-3 fatty acids normalize BDNF levels, reduce oxidative damage, and counteract learning disability after traumatic brain injury in rats. J Neurotrauma. (2004) 21:1457–67. doi: 10.1089/neu.2004.21.1457, PMID: 15672635

[32] Thiyagarajan M Sharma SS. Neuroprotective effect of curcumin in middle cerebral artery occlusion induced focal cerebral ischemia in rats. Life Sci. (2004) 74:969–85. doi: 10.1016/j.lfs.2003.06.042, PMID: 14672754

[33] Li WSNC . Curcumin by down-regulating NF-kB and elevating Nrf2, reduces brain edema and neurological dysfunction after cerebral I/R. Microvasc Res. (2016) 106:117–27. doi: 10.1016/j.mvr.2015.12.008 26686249

[34] González-Reyes S Guzmán-Beltrán S Medina-Campos ON Pedraza-Chaverri J. Curcumin pretreatment induces Nrf2 and an antioxidant response and prevents hemin-induced toxicity in primary cultures of cerebellar granule neurons of rats. Oxidative Med Cell Longev. (2013) 2013:1–14. doi: 10.1155/2013/801418, PMID: 24454990 PMC3885319

[35] Cui X Song H Su J. Curcumin attenuates hypoxic-ischemic brain injury in neonatal rats through induction of nuclear factor erythroid-2-related factor 2 and heme oxygenase-1. Exp Ther Med. (2017) 14:1512–8. doi: 10.3892/etm.2017.4683, PMID: 28781627 PMC5526188

[36] Tu ZS Wang Q Sun DD Dai F Zhou B. Design, synthesis, and evaluation of curcumin derivatives as Nrf2 activators and cytoprotectors against oxidative death. Eur J Med Chem. (2017) 134:72–85. doi: 10.1016/j.ejmech.2017.04.008, PMID: 28399452

[37] Xie YL Chu JG Jian XM Dong JZ Wang LP Li GX . Curcumin attenuates lipopolysaccharide/d-galactosamine-induced acute liver injury by activating Nrf2 nuclear translocation and inhibiting NF-kB activation. Biomed Pharmacother. (2017) 91:70–7. doi: 10.1016/j.biopha.2017.04.070, PMID: 28448872

[38] Wu A Ying Z Gomez-Pinilla F. Omega-3 fatty acids supplementation restores mechanisms that maintain brain homeostasis in traumatic brain injury. J Neurotrauma. (2007) 24:1587–95. doi: 10.1089/neu.2007.0313 17970622

[39] Ko HR Ahn SY Chang YS Hwang I Yun T Sung DK . Human UCB-MSCs treatment upon intraventricular hemorrhage contributes to attenuate hippocampal neuron loss and circuit damage through BDNF-CREB signaling. Stem Cell Res Ther. (2018) 9:326. doi: 10.1186/s13287-018-1052-5, PMID: 30463591 PMC6249960

[40] Liang J Deng G Huang H. The activation of BDNF reduced inflammation in a spinal cord injury model by TrkB/p38 MAPK signaling. Exp Ther Med. (2019) 17:1688–96. doi: 10.3892/etm.2018.7109, PMID: 30783437 PMC6364215

[41] Shi LY Zhang L Li H Liu TL Lai JC Wu ZB . Protective effects of curcumin on acrolein-induced neurotoxicity in HT22 mouse hippocampal cells. Pharmacol Rep. (2018) 70:1040–6. doi: 10.1016/j.pharep.2018.05.006, PMID: 30144665

[42] Yang J Song S Li J Liang T. Neuroprotective effect of curcumin on hippocampal injury in 6-OHDA-induced Parkinson's disease rat. Pathol Res Pract. (2014) 210:357–62. doi: 10.1016/j.prp.2014.02.005, PMID: 24642369

[43] Wu A Ying Z Gomez-Pinilla F. The salutary effects of DHA dietary supplementation on cognition, neuroplasticity, and membrane homeostasis after brain trauma. J Neurotrauma. (2011b) 28:2113–22. doi: 10.1089/neu.2011.1872, PMID: 21851229 PMC3191367

[44] Hariri AR Goldberg TE Mattay VS Kolachana BS Callicott JH Egan MF . Brain-derived neurotrophic factor val66met polymorphism affects human memory-related hippocampal activity and predicts memory performance. J Neurosci. (2003) 23:6690–4. doi: 10.1523/JNEUROSCI.23-17-06690.2003, PMID: 12890761 PMC6740735

[45] Ying SW Futter M Rosenblum K Webber MJ Hunt SP Bliss TV . Brain-derived neurotrophic factor induces long-term potentiation in intact adult hippocampus: requirement for ERK activation coupled to CREB and upregulation of arc synthesis. J Neurosci. (2002) 22:1532–40. doi: 10.1523/JNEUROSCI.22-05-01532.2002, PMID: 11880483 PMC6758896

[46] Qin ZH Wang Y Kegel KB Kazantsev A Apostol BL Thompson LM . Autophagy regulates the processing of amino terminal huntingtin fragments. Hum Mol Genet. (2003) 12:3231–44. doi: 10.1093/hmg/ddg346, PMID: 14570716

[47] Sheng R Zhang L Han R Liu X Gao B Qin Z. Autophagy activation is associated with neuroprotection in a rat model of focal cerebral ischemic preconditioning. Autophagy. (2010) 6:482–94. doi: 10.4161/auto.6.4.11737, PMID: 20400854

[48] Tanaka K Komatsu M Waguri S Chiba T Murata S Iwata J . Loss of autophagy in the central nervous system causes neurodegeneration in mice. Nature. (2006) 441:880–4. doi: 10.1038/nature04723 16625205

[49] Tanida I Ueno T Kominami E. LC3 and autophagy. Totowa, NJ: Humana Press (2008). 445:77–88. 10.1007/978-1-59745-157-4_4 18425443

[50] Chen J Wang Q Yin FQ Zhang W Yan LH Li L. MTRR silencing inhibits growth and cisplatin resistance of ovarian carcinoma via inducing apoptosis and reducing autophagy. Am J Transl Res. (2015) 7:1510–27. PMID: 26550452 PMC4626414

[51] Cheng KK Yeung CF Ho SW Chow SF Chow AHL Baum L. Highly stabilized curcumin nanoparticles tested in an in vitro blood–brain barrier model and in Alzheimer’s disease Tg2576 mice. AAPS J. (2013) 15:324–36. doi: 10.1208/s12248-012-9444-4 23229335 PMC3675736

[52] Julien O Wells JA. Caspases and their substrates. Cell Death Differ. (2017) 24:1380–9. doi: 10.1038/cdd.2017.44, PMID: 28498362 PMC5520456

[53] Wu H Lu D Jiang H Xiong Y Qu C Li B . Simvastatin-mediated upregulation of VEGF and BDNF, activation of the PI3K/Akt pathway, and increase of neurogenesis are associated with therapeutic improvement after traumatic brain injury. J Neurotrauma. (2008) 25:130–9. doi: 10.1089/neu.2007.0369, PMID: 18260796

[54] Ashkenazi A Dixit VM. Death receptors: signaling and modulation. Science. (1998) 281:1305–8. doi: 10.1126/science.281.5381.1305 9721089

[55] Popgeorgiev N Jabbour L Gillet G. Subcellular localization and dynamics of the Bcl-2 family of proteins. Front Cell Dev Biol. (2018) 6:13. doi: 10.3389/fcell.2018.00013, PMID: 29497611 PMC5819560

[56] Xia C Cai Y Li S Yang J Xiao G. Curcumin increases HSP70 expression in primary rat cortical neuronal apoptosis induced by gp120 V3 loop peptide. Neurochem Res. (2015) 40:1996–2005. doi: 10.1007/s11064-015-1695-x, PMID: 26294283

