# Supplementary material for: Curcumin in the treatment of inflammation and oxidative stress responses in traumatic brain injury: a systematic review and meta-analysis
Source: Front Neurol. 2024 May 10;15:1380353. doi: 10.3389/fneur.2024.1380353 (PMC11116723; doi:10.3389/fneur.2024.1380353)
Supplement: Supplementary file 1 [file Data_Sheet_1.pdf]

Inflammatory factors

1.IL-1β

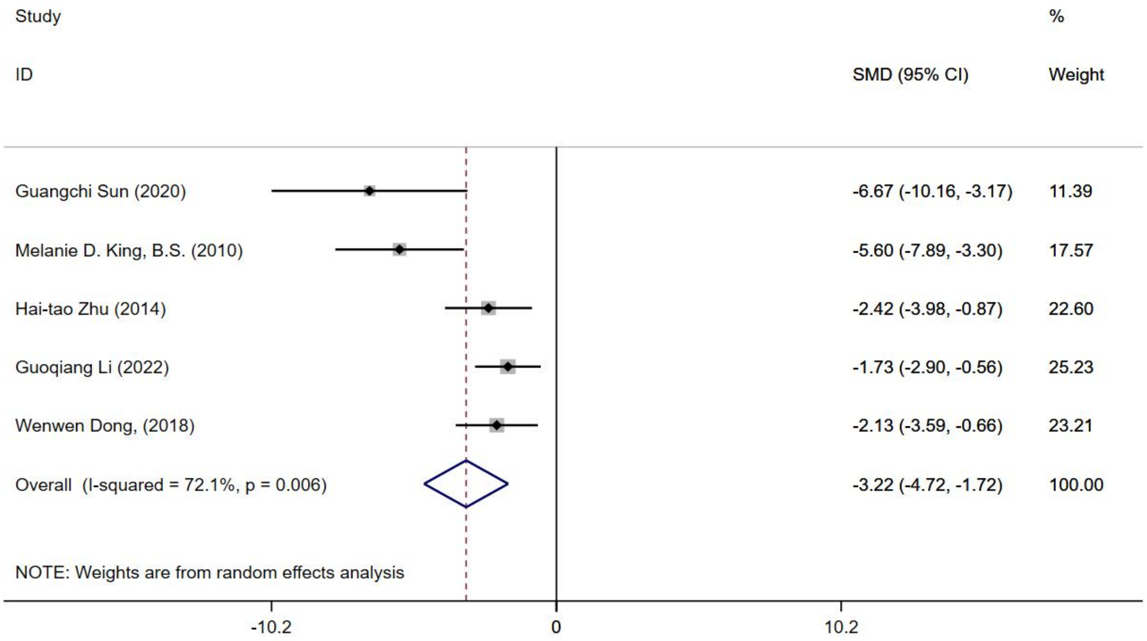

2.IL-6

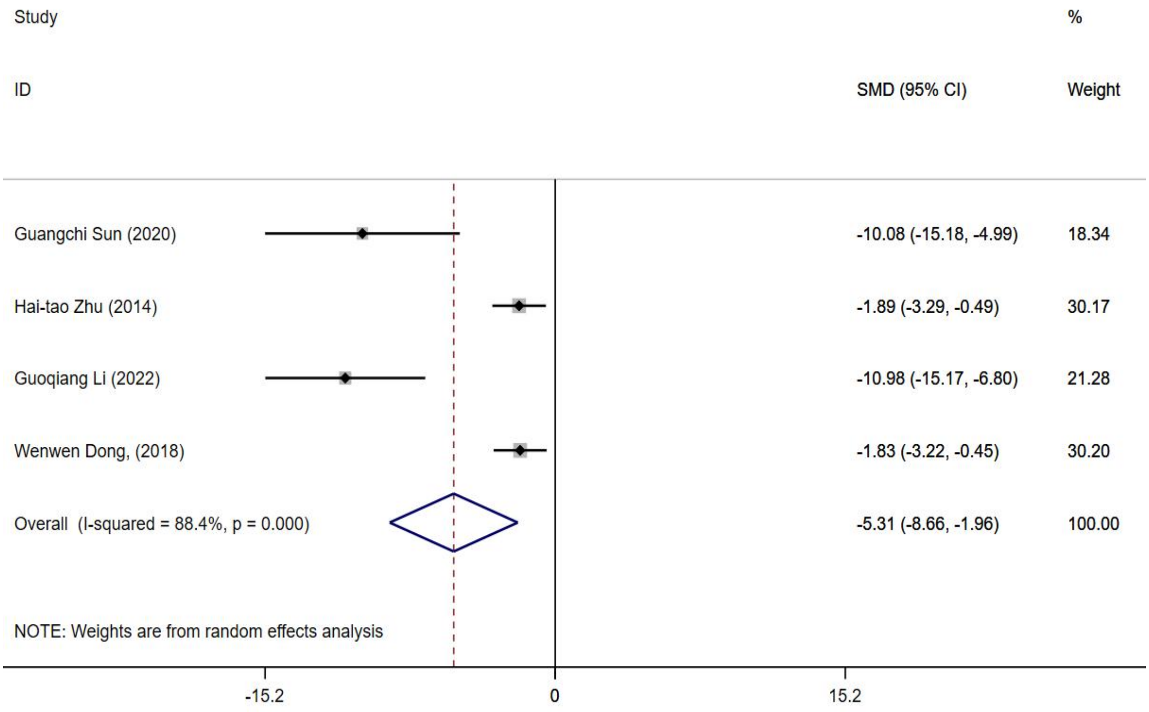

3. TNF-α

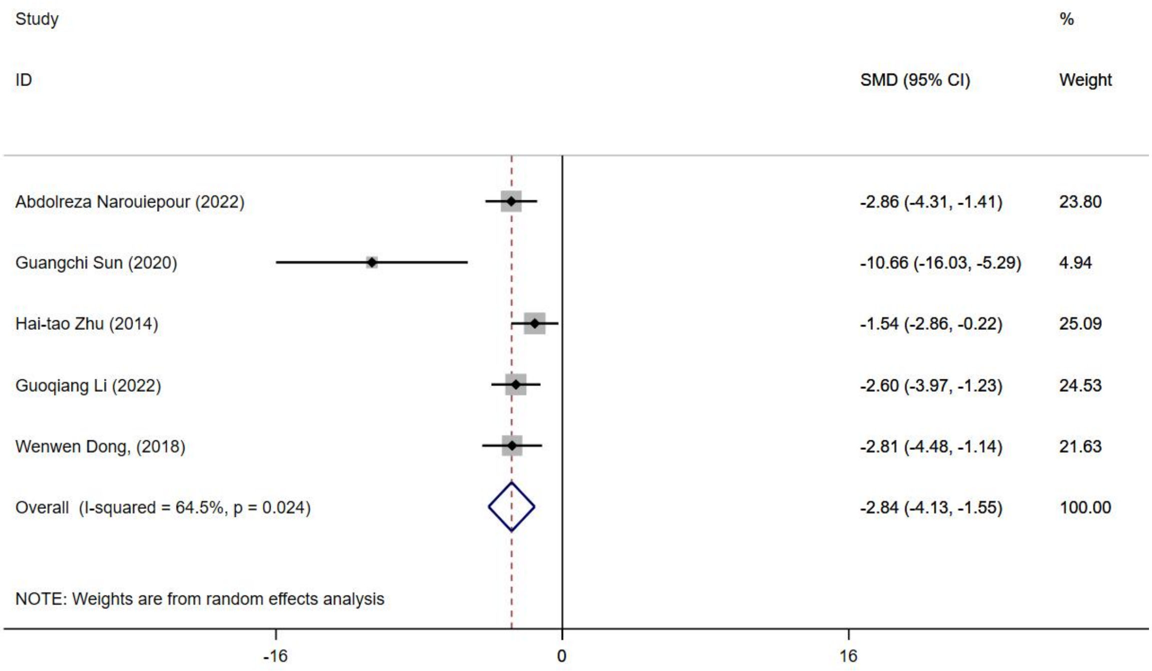

Oxidative stress factor

1.SOD

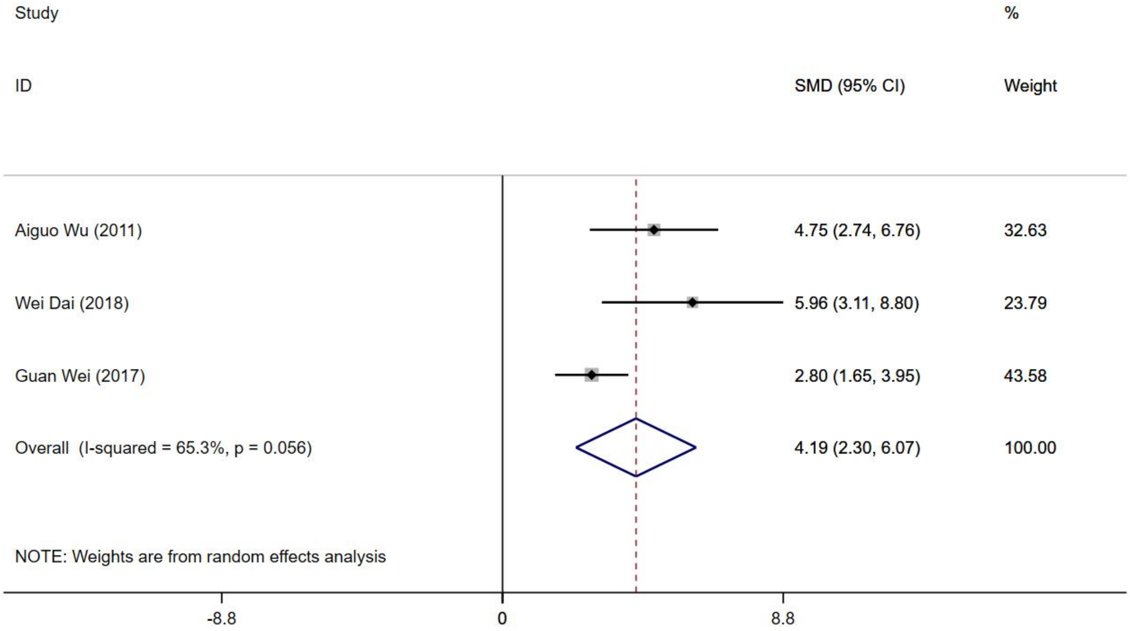

2.Sir2

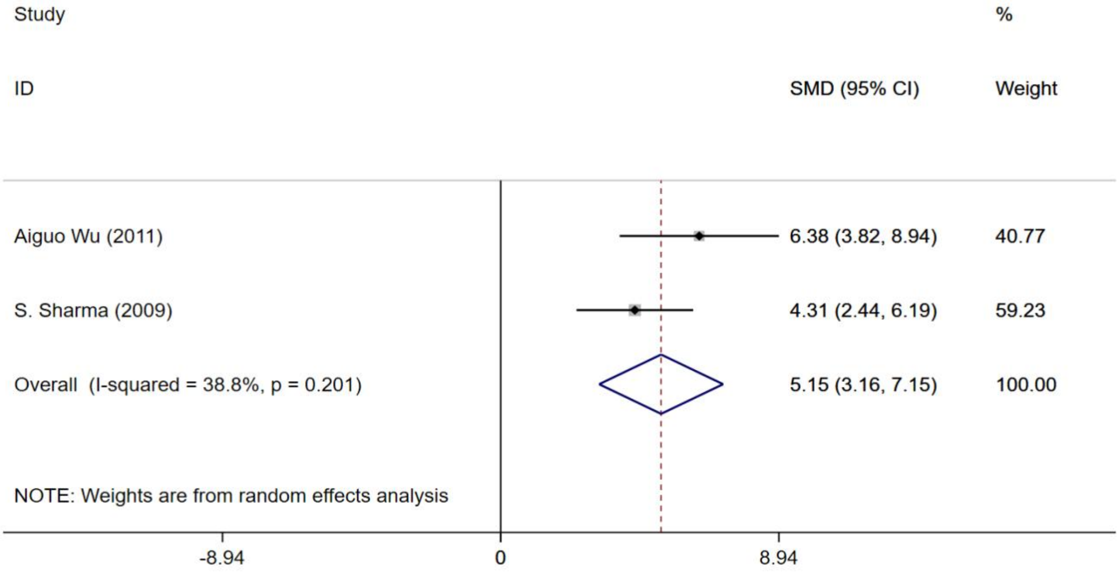
Study
ID
%
SMD (95% CI)
Weight
Aiguo Wu (2011)
6.38 (3.82, 8.94)
40.77
S. Sharma (2009)
4.31 (2.44, 6.19)
59.23
Overall (I-squared = 38.8%, p = 0.201)
5.15 (3.16, 7.15)
100.00
NOTE: Weights are from random effects analysis
-8.94
0
8.94

3.GPx

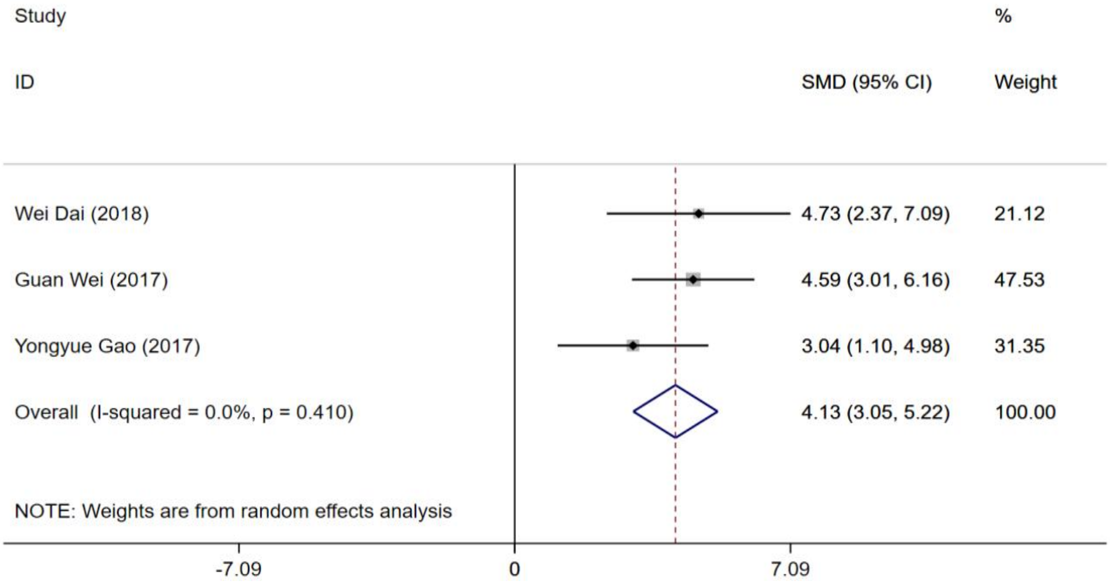
Study
ID
%
SMD (95% CI)
Weight
Wei Dai (2018)
4.73 (2.37, 7.09)
21.12
Guan Wei (2017)
4.59 (3.01, 6.16)
47.53
Yongyue Gao (2017)
3.04 (1.10, 4.98)
31.35
Overall (I-squared = 0.0%, p = 0.410)
4.13 (3.05, 5.22)
100.00
NOTE: Weights are from random effects analysis
-7.09
0
7.09

4.Nrf2

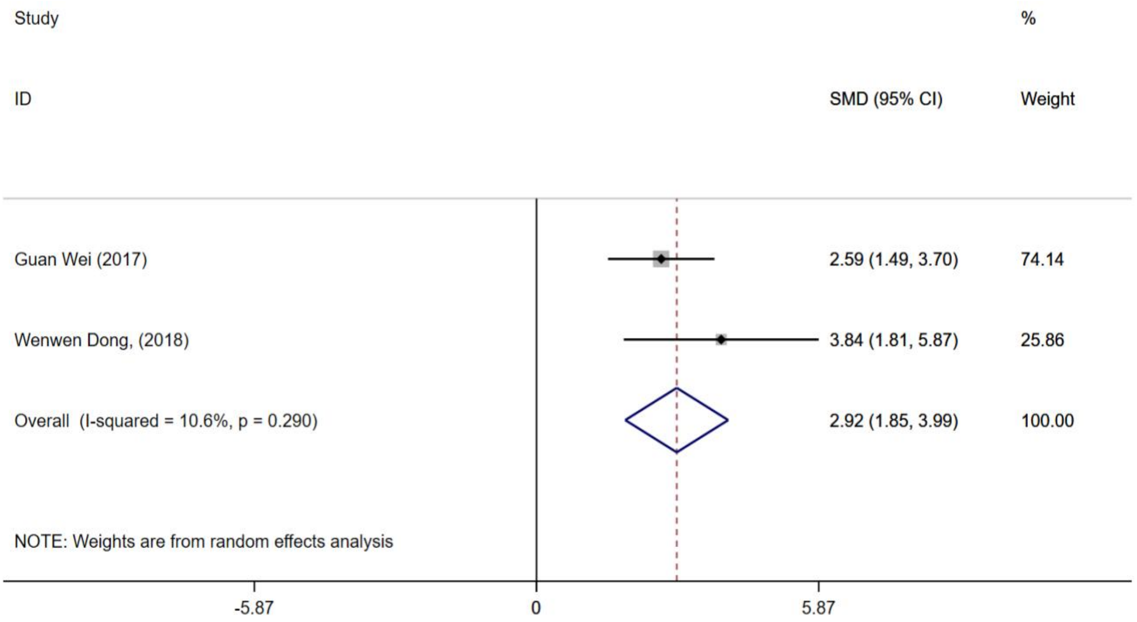
Study
%
ID
SMD (95% CI)
Weight
Guan Wei (2017)
2.59 (1.49, 3.70)
74.14
Wenwen Dong, (2018)
3.84 (1.81, 5.87)
25.86
Overall (I-squared = 10.6%, p = 0.290)
2.92 (1.85, 3.99)
100.00
NOTE: Weights are from random effects analysis
-5.87
0
5.87

5.MDA

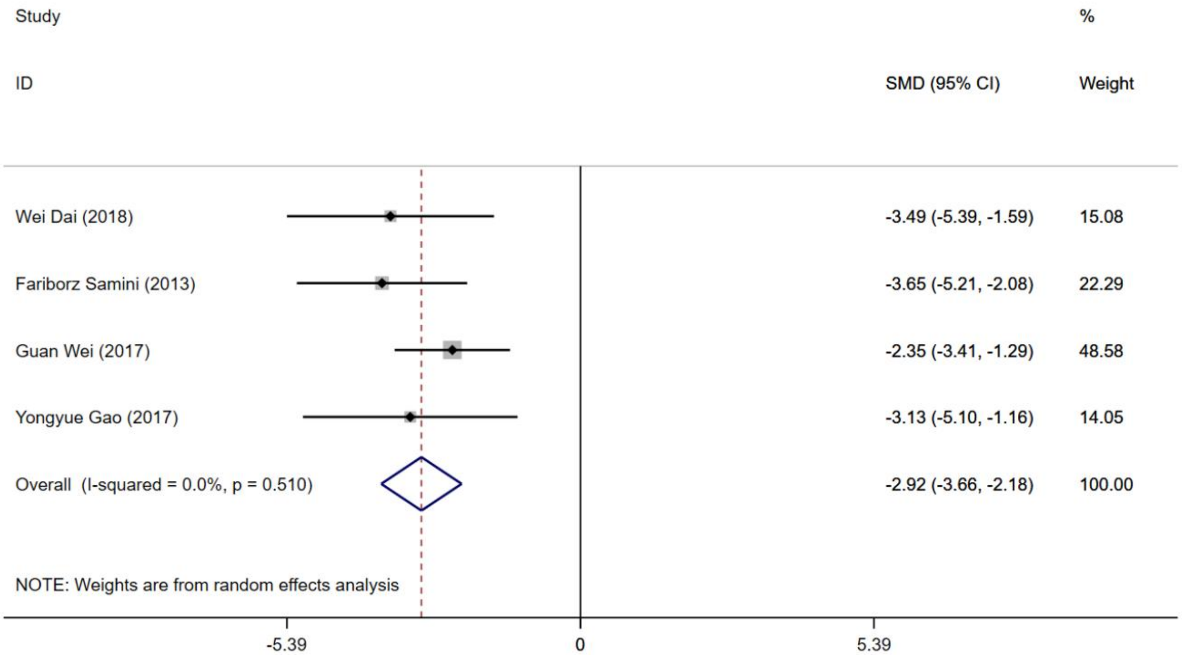
Study
%
ID
SMD (95% CI)
Weight
Wei Dai (2018)
-3.49 (-5.39, -1.59)
15.08
Fariborz Samini (2013)
-3.65 (-5.21, -2.08)
22.29
Guan Wei (2017)
-2.35 (-3.41, -1.29)
48.58
Yongyue Gao (2017)
-3.13 (-5.10, -1.16)
14.05
Overall (I-squared = 0.0%, p = 0.510)
-2.92 (-3.66, -2.18)
100.00
NOTE: Weights are from random effects analysis
-5.39
0
5.39

6.4-HNE

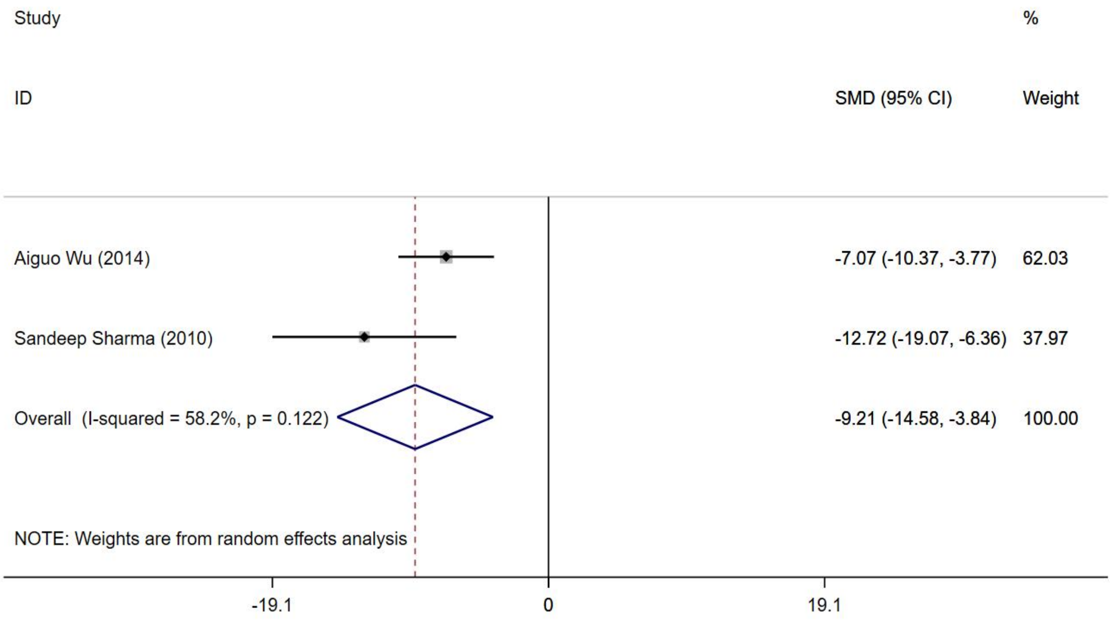

7.Protein carbonyl level

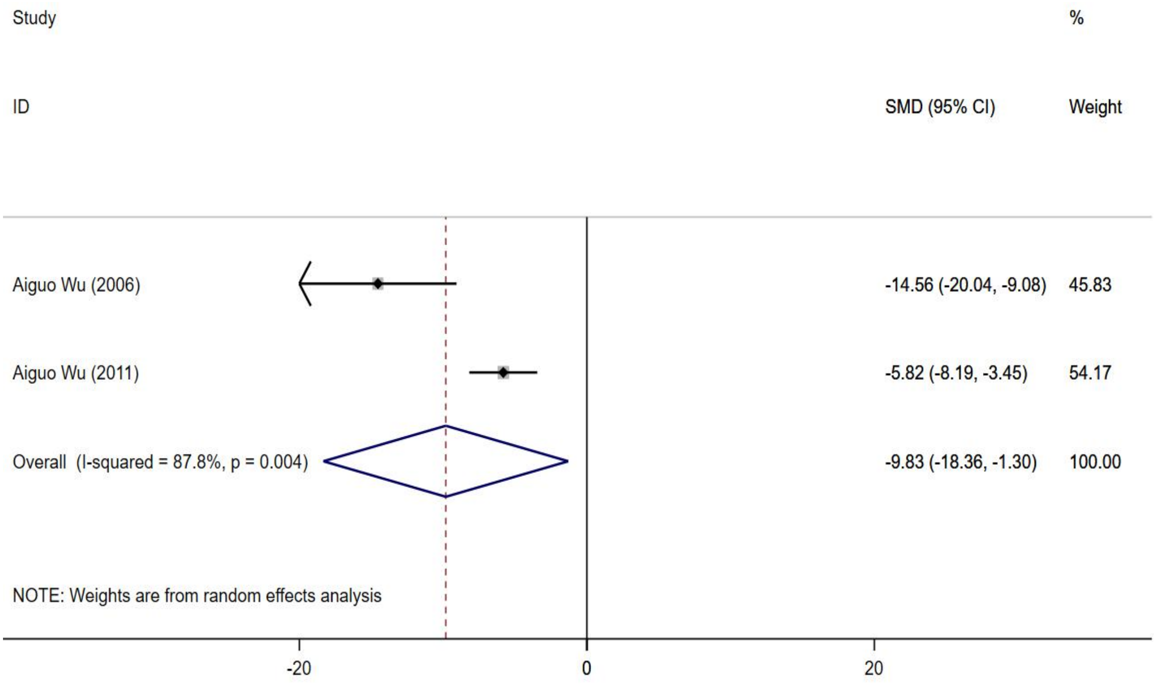

Neuroprotective factor

1.Brain water content

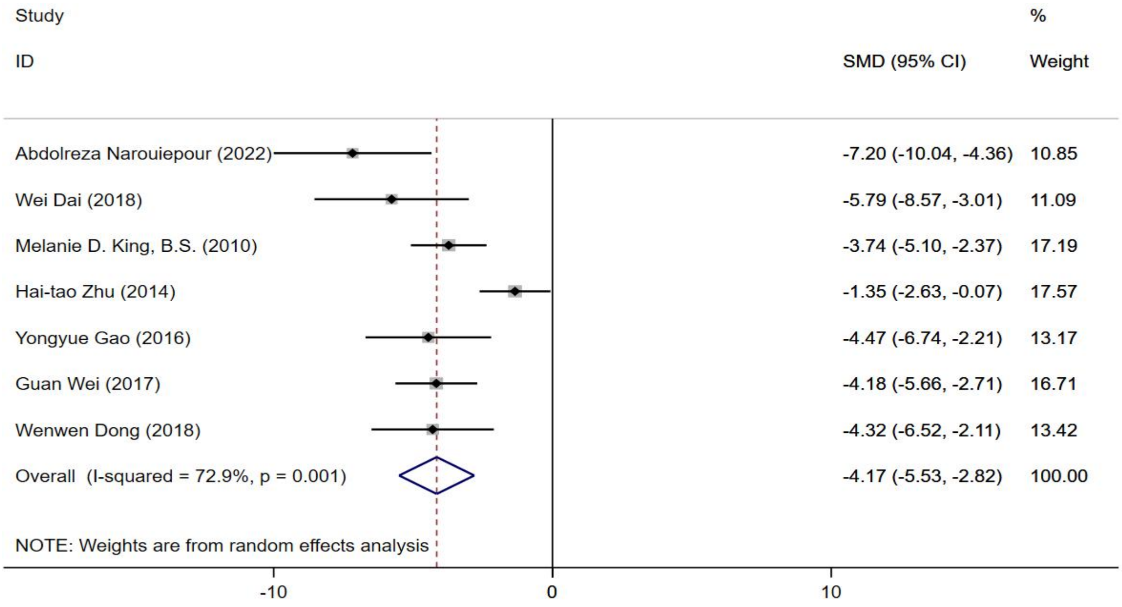

2.mNSS

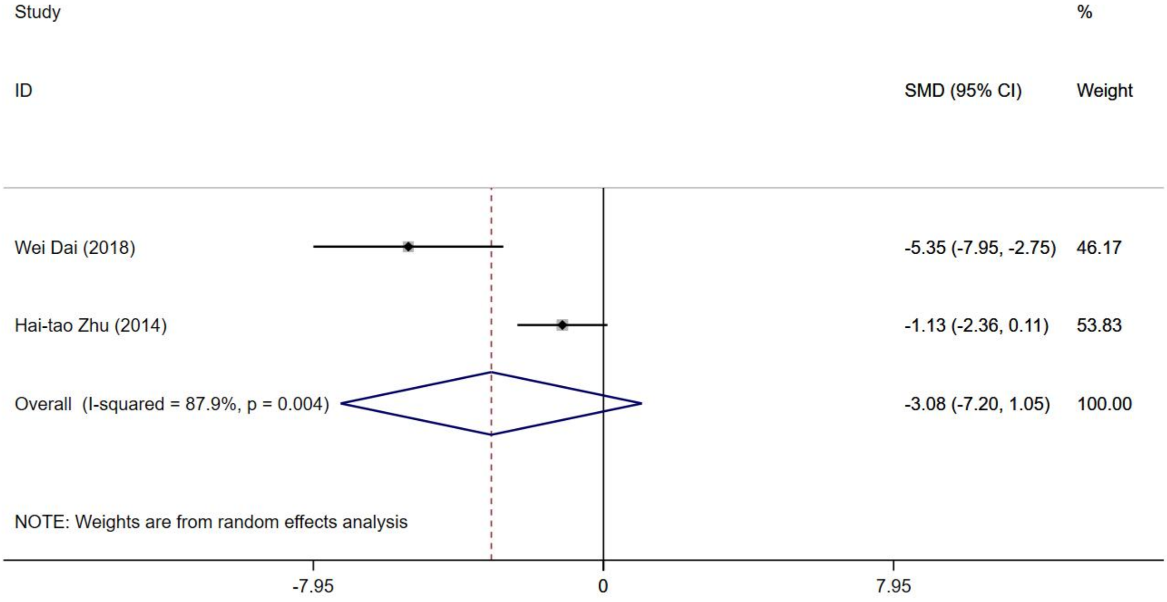

3.SynapsinI

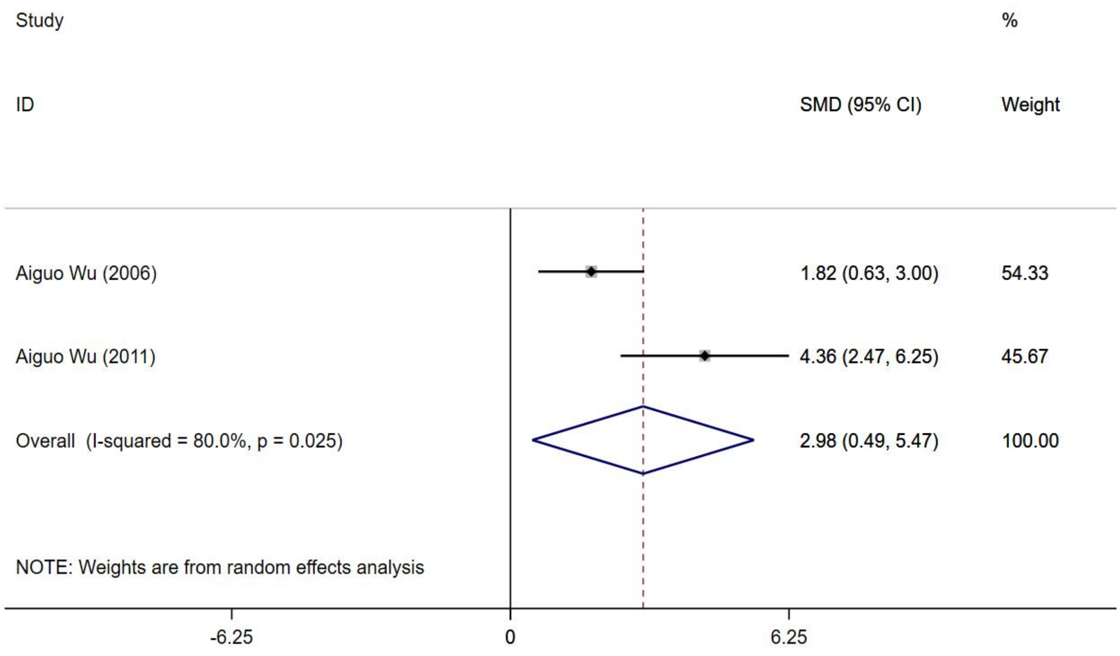

4.BDNF

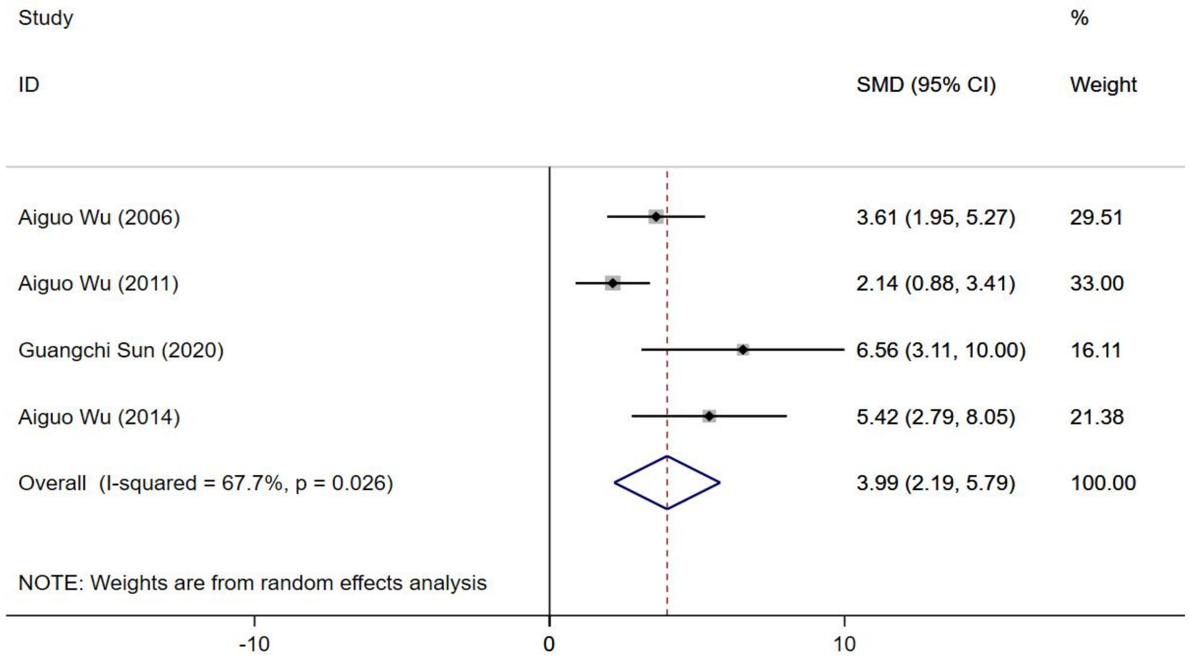

5.CREB

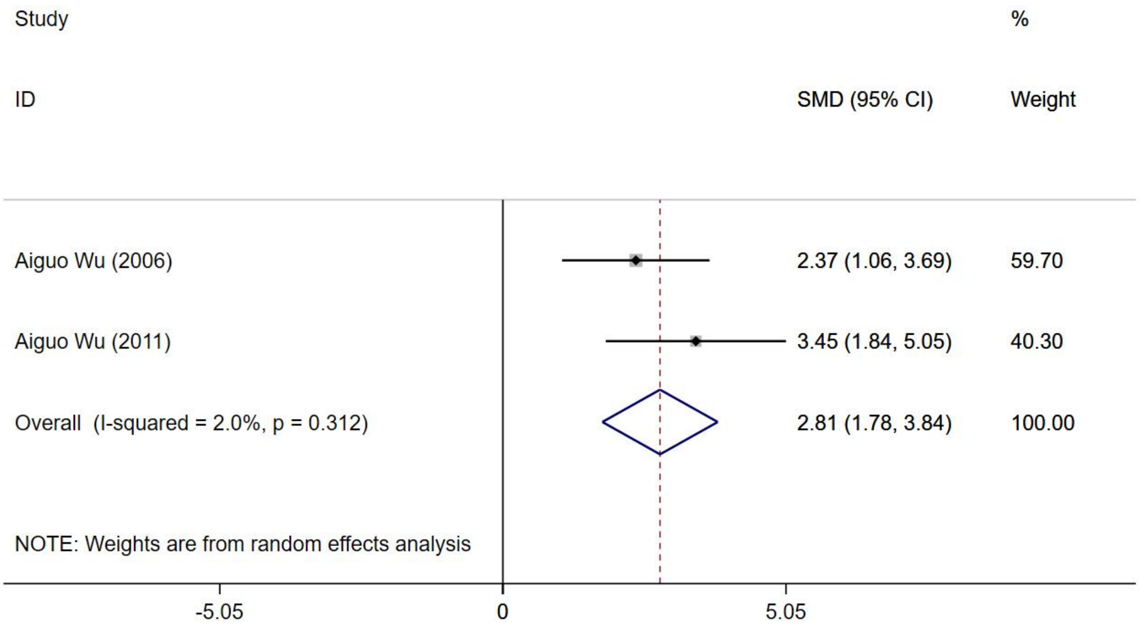

Autophagy and apoptosis factors

1.Bcl-2

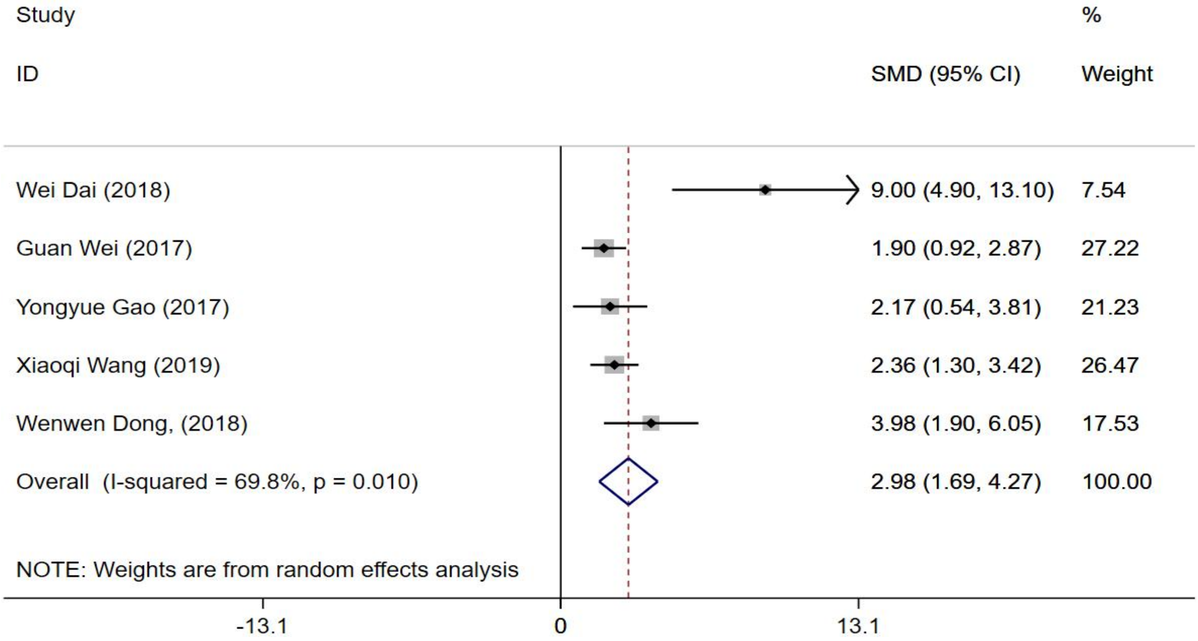

2.Beclin-1

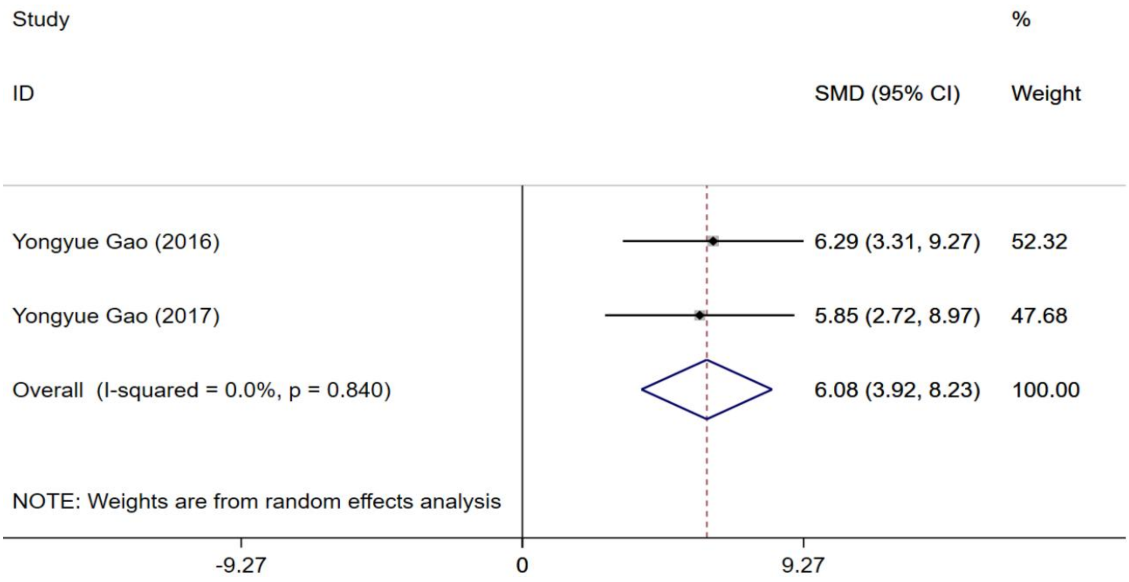

3.Caspase-3

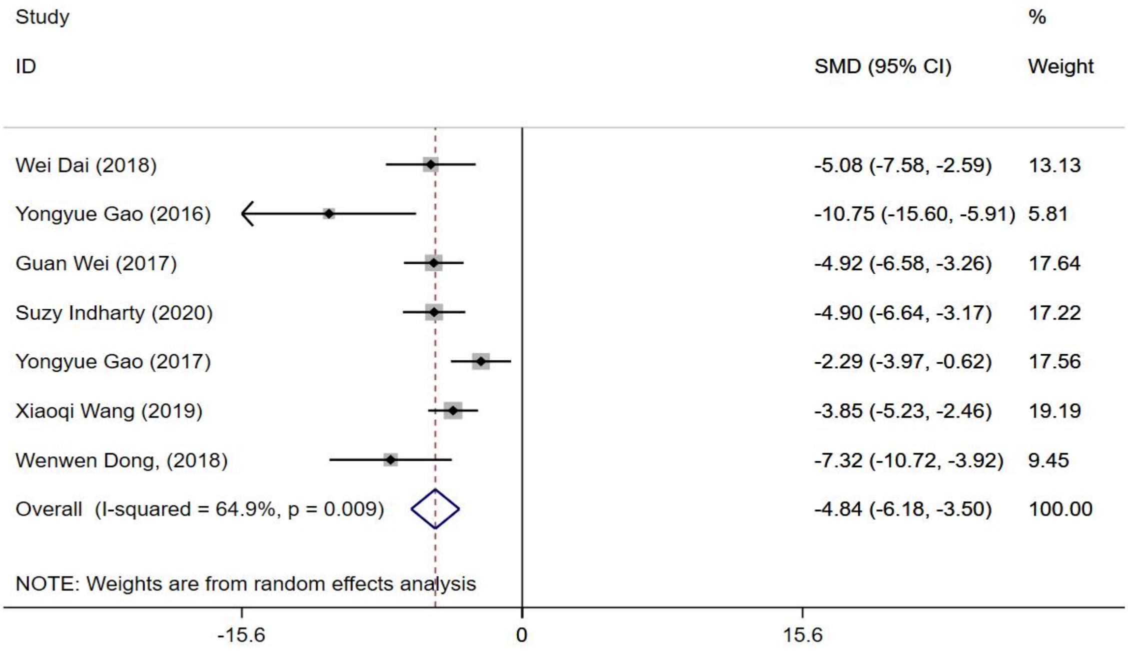

4.Apoptosis index

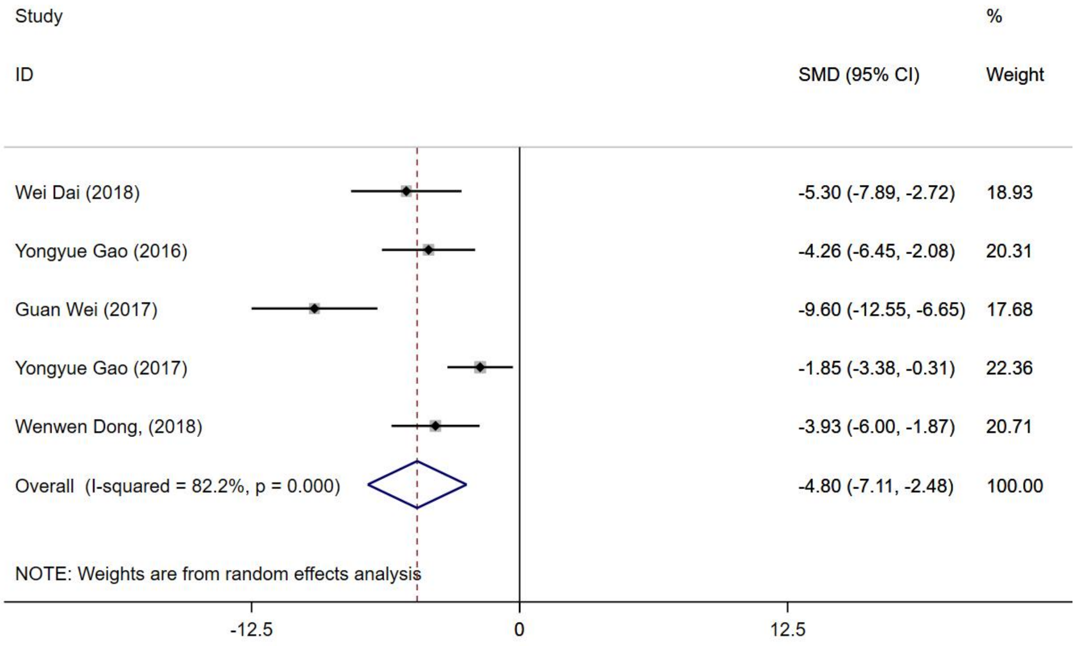

5.P62

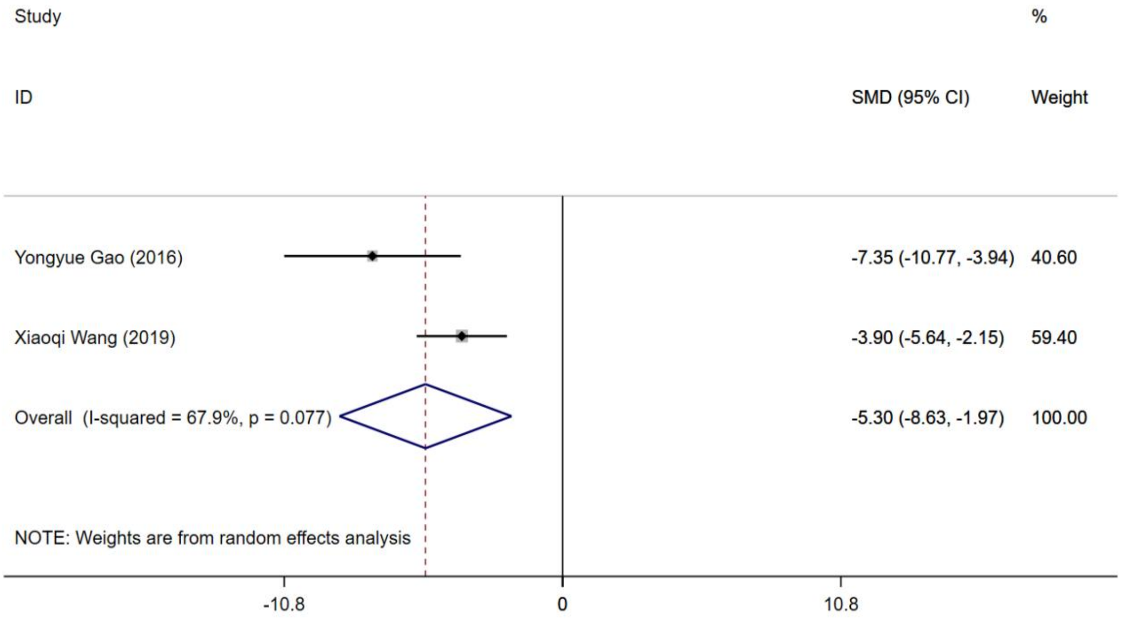

IL-1β, Interleukin-1β; IL-6, Interleukin-6; TNF-α, Tumor necrosis factor-α; SOD, Superoxide dismutase; Sir2, Silent information regulator 2; GPx, Glutathione peroxidase; Nrf2, NF-E2-related factor; MDA, Malondialdehyde; 4-HNE, 4-hydroxynonenal; mNSS, Modified neurological severity score; BDNF, Brain-derived neurotrophic factor; CREB, Cyclic AMP-response element-binding protein; Bcl-2, B-cell lymphoma/leukemia 2; P62, Autophagy associated protein.
