# Supplementary material for: Curcumin in the treatment of inflammation and oxidative stress responses in traumatic brain injury: a systematic review and meta-analysis
Source: Front Neurol. 2024 May 10;15:1380353. doi: 10.3389/fneur.2024.1380353 (PMC11116723; doi:10.3389/fneur.2024.1380353)
Supplement: Supplementary file 2 [file Data_Sheet_2.pdf]

Inflammatory factors

1.IL-1β

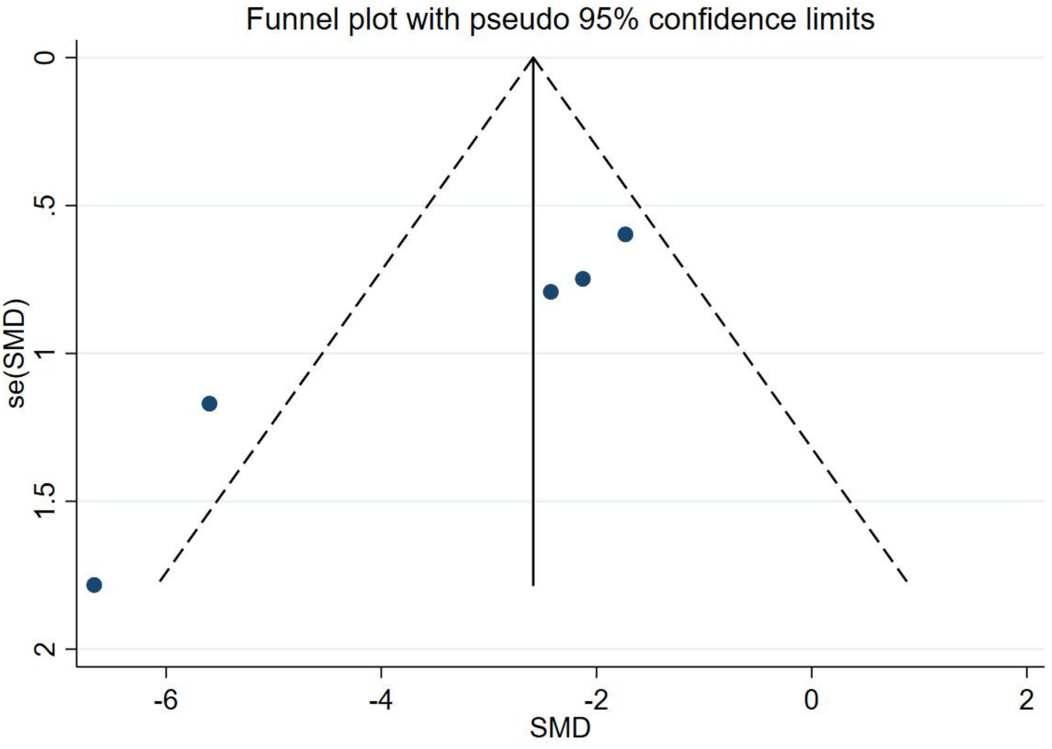

2.IL-6

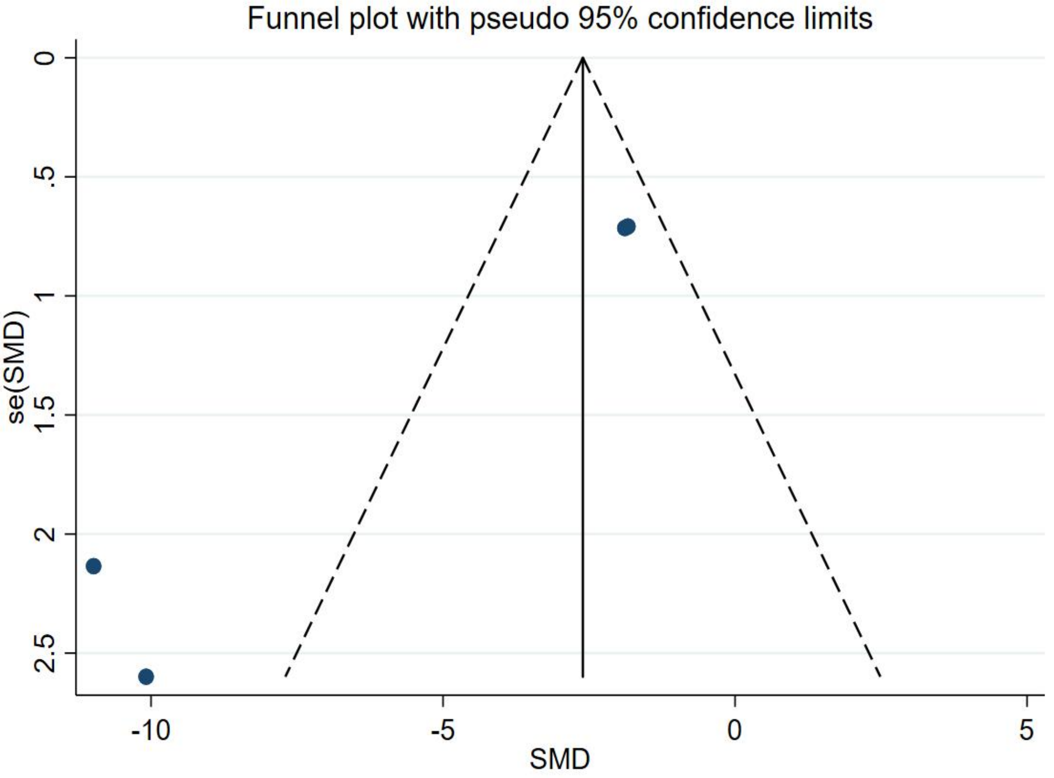

3. TNF-α

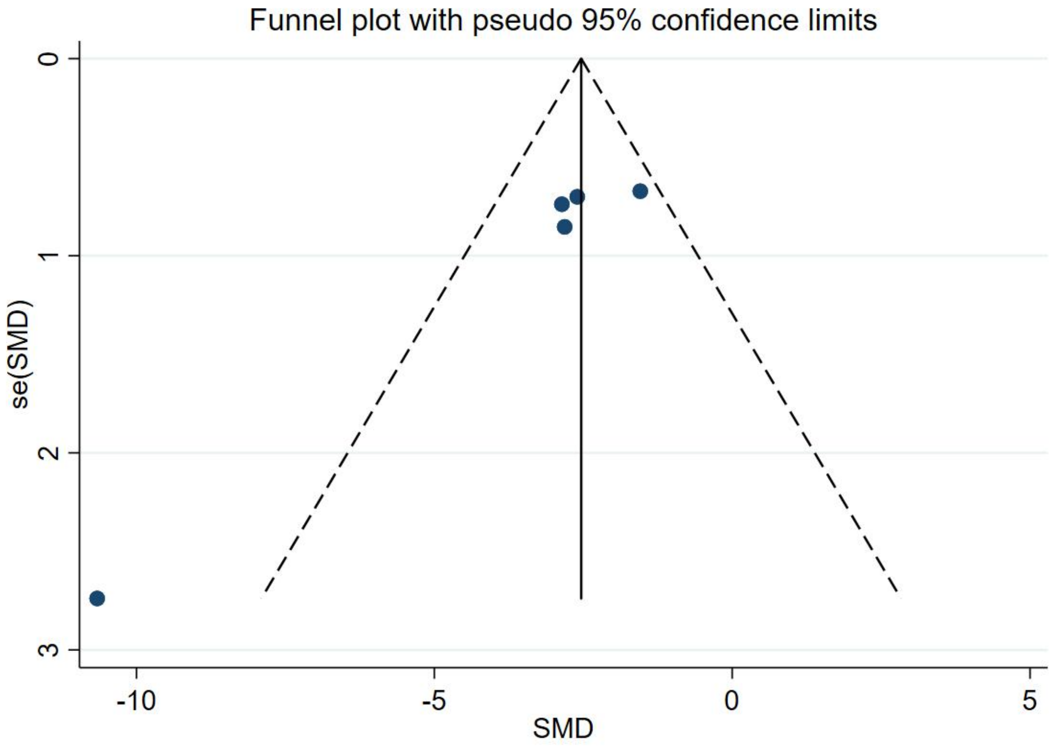

Oxidative stress factor

1.SOD

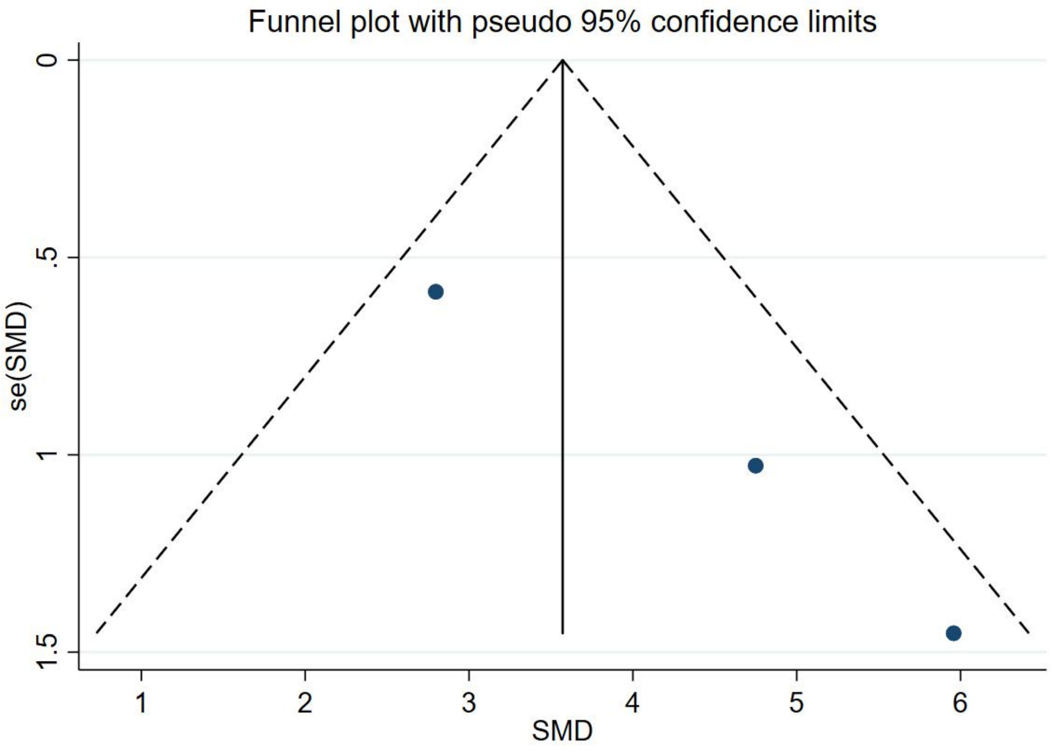

2.Sir2

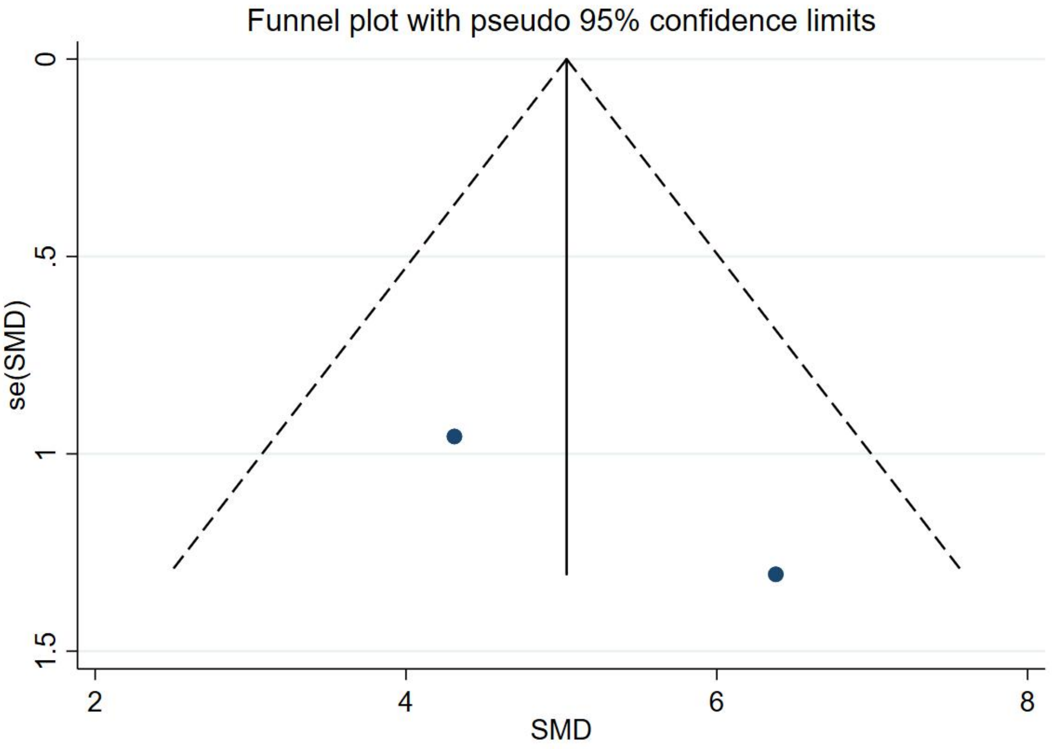

Funnel plot with pseudo 95% confidence limits
0
.5
1
1.5
se(SMD)
2
4
6
8
SMD

3.GPx

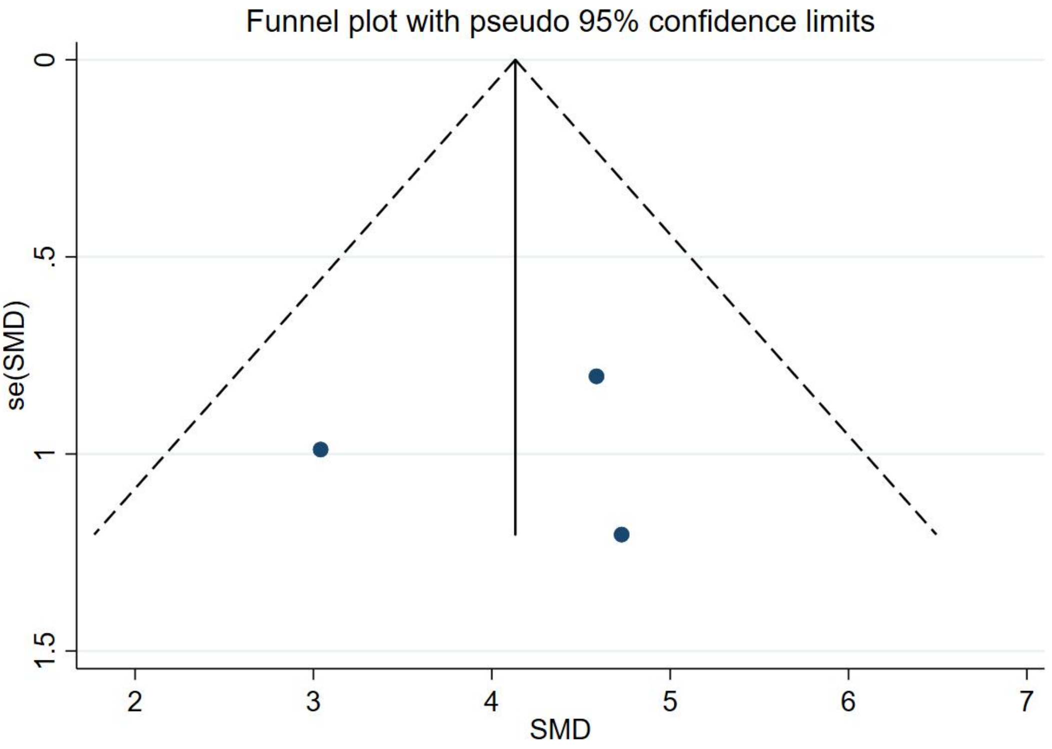

Funnel plot with pseudo 95% confidence limits
0
.5
1
1.5
se(SMD)
2
3
4
5
6
7
SMD

4.Nrf2

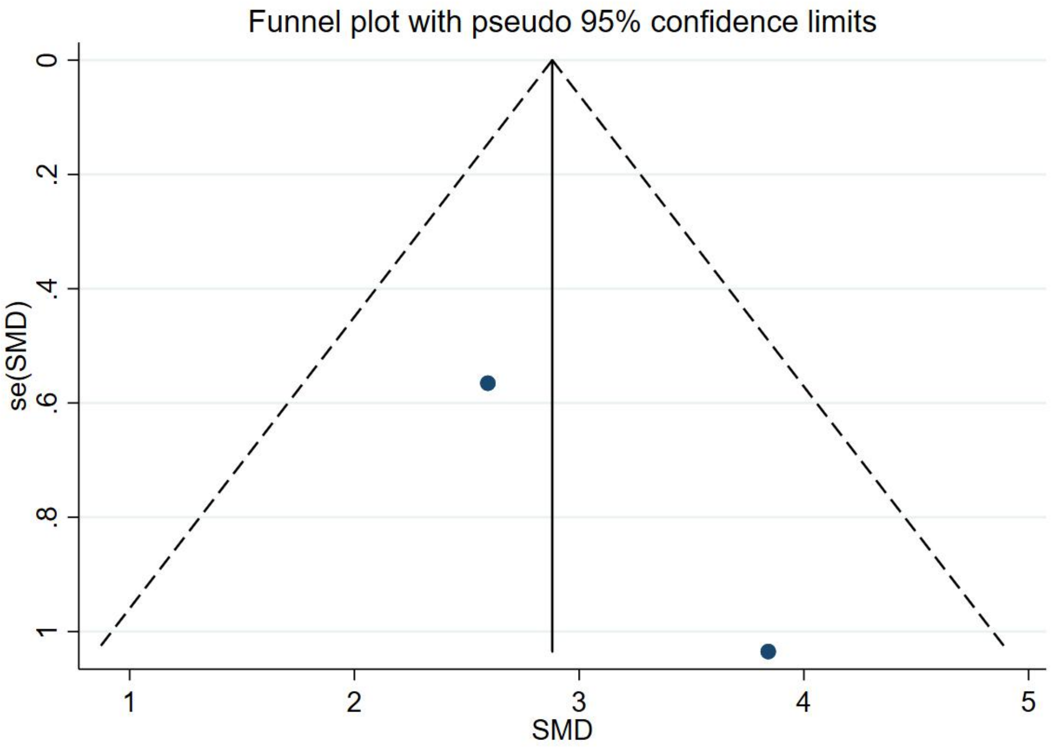
Funnel plot with pseudo 95% confidence limits
se(SMD)
0
.2
.4
.6
.8
1
1
2
3
4
5
SMD

5.MDA

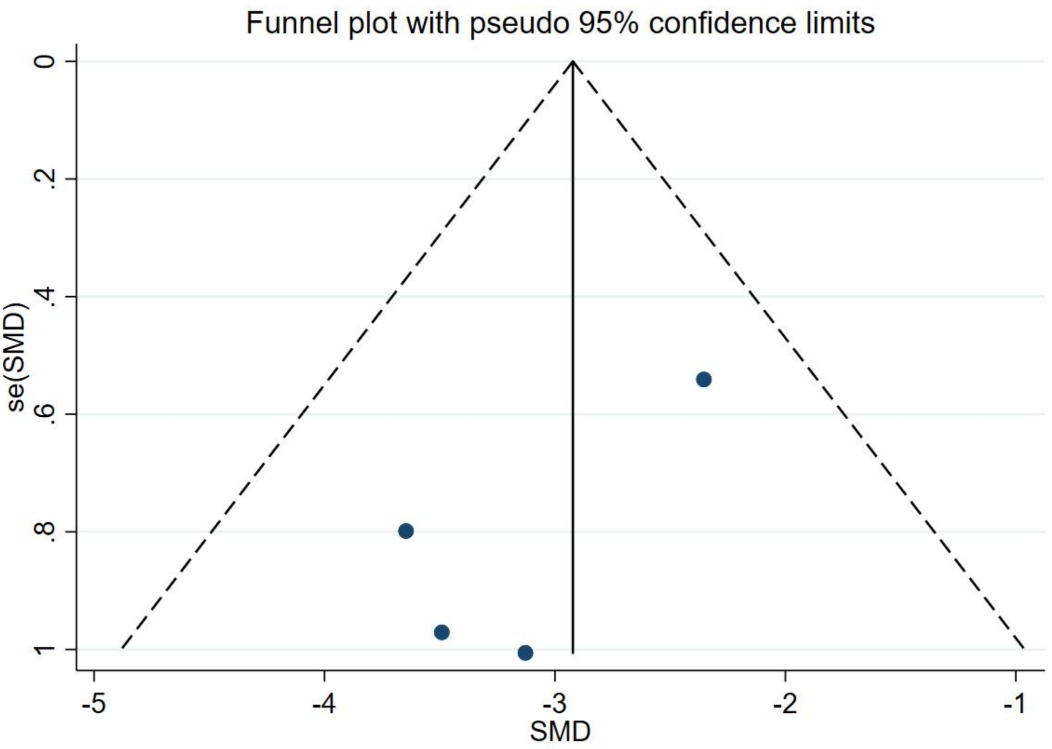
Funnel plot with pseudo 95% confidence limits
se(SMD)
0
.2
.4
.6
.8
1
-5
-4
-3
-2
-1
SMD

6.4-HNE

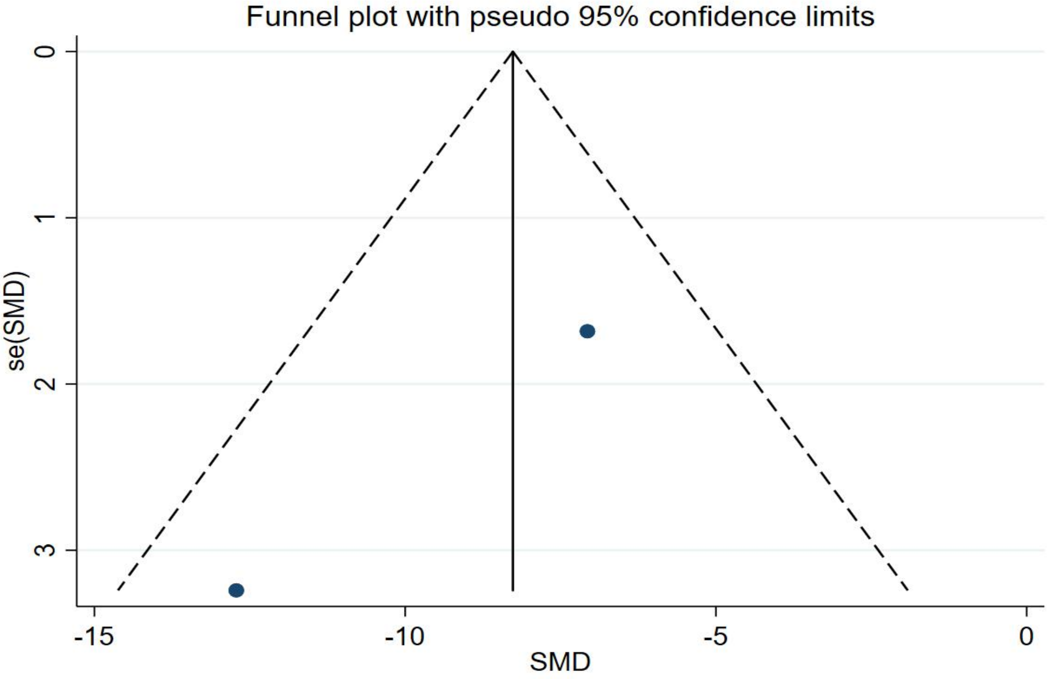

7.Protein carbonyllevel

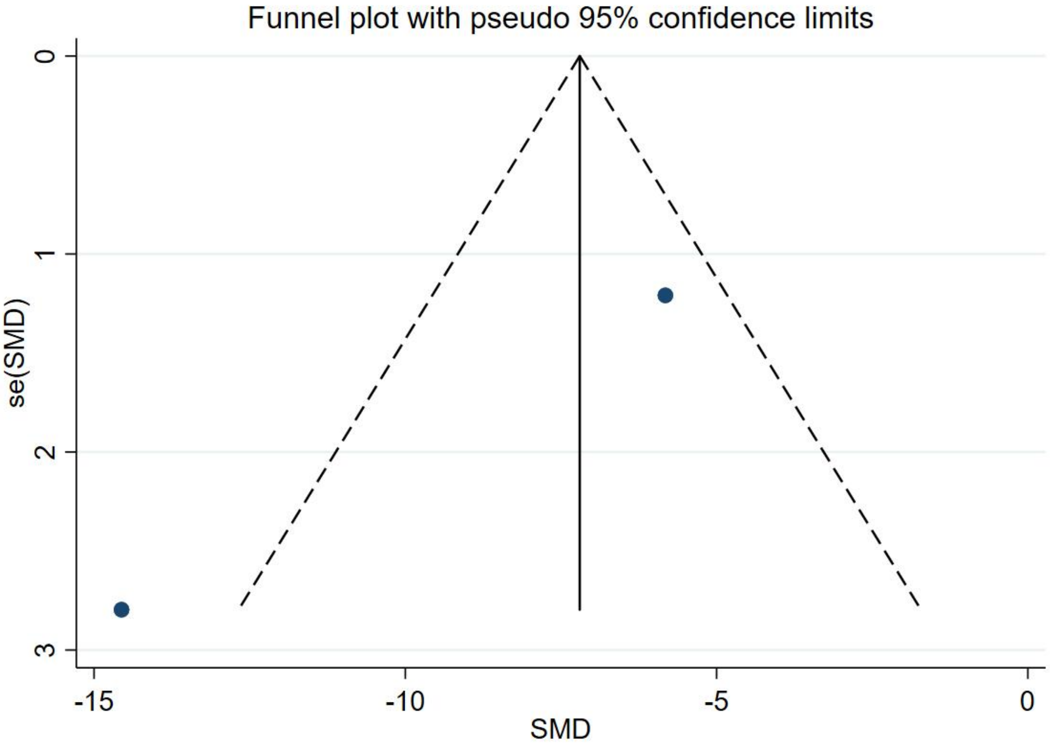

Neuroprotective factor

1.Brain water content

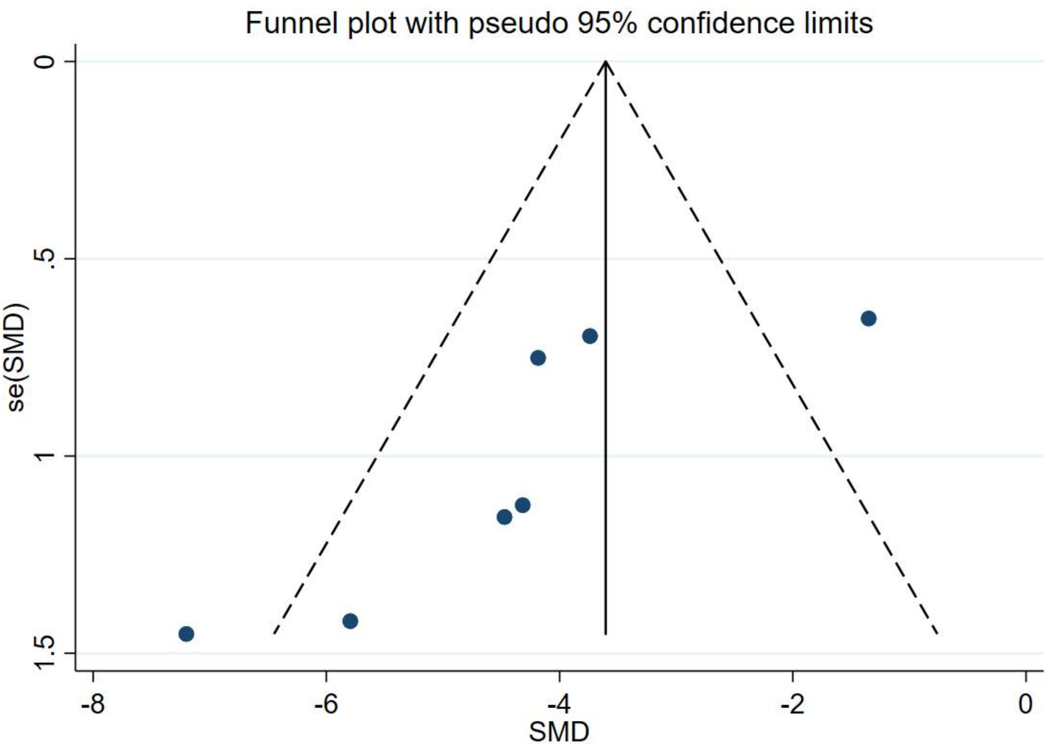

2.mNSS

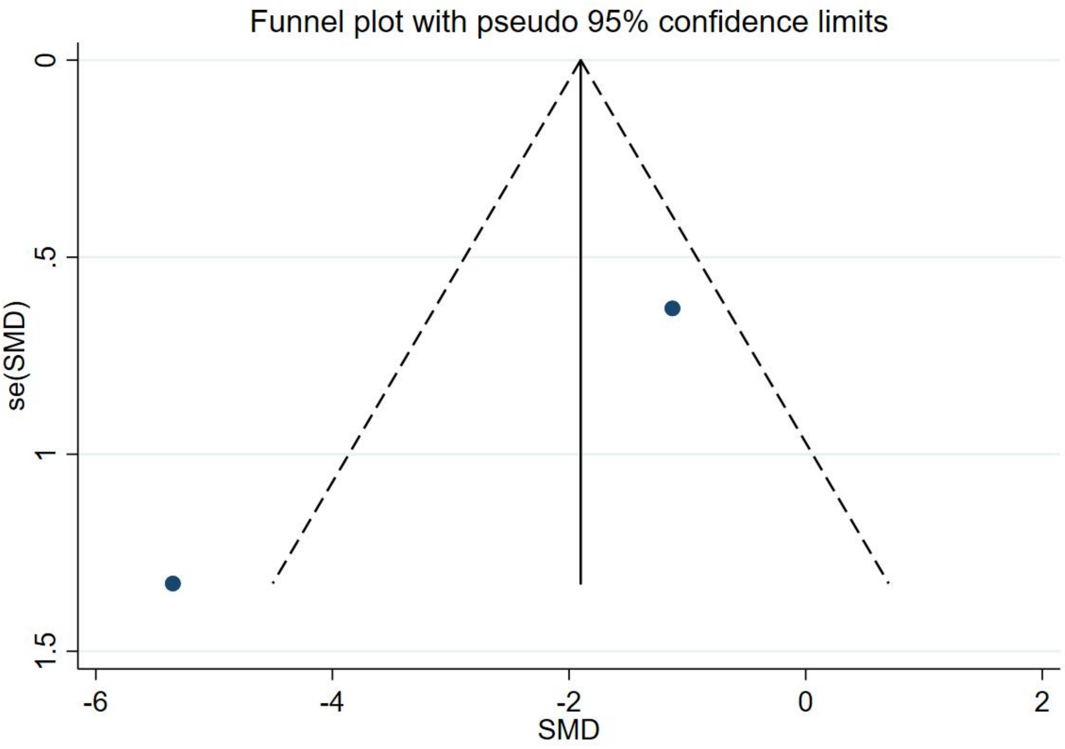

3.SynapsinI

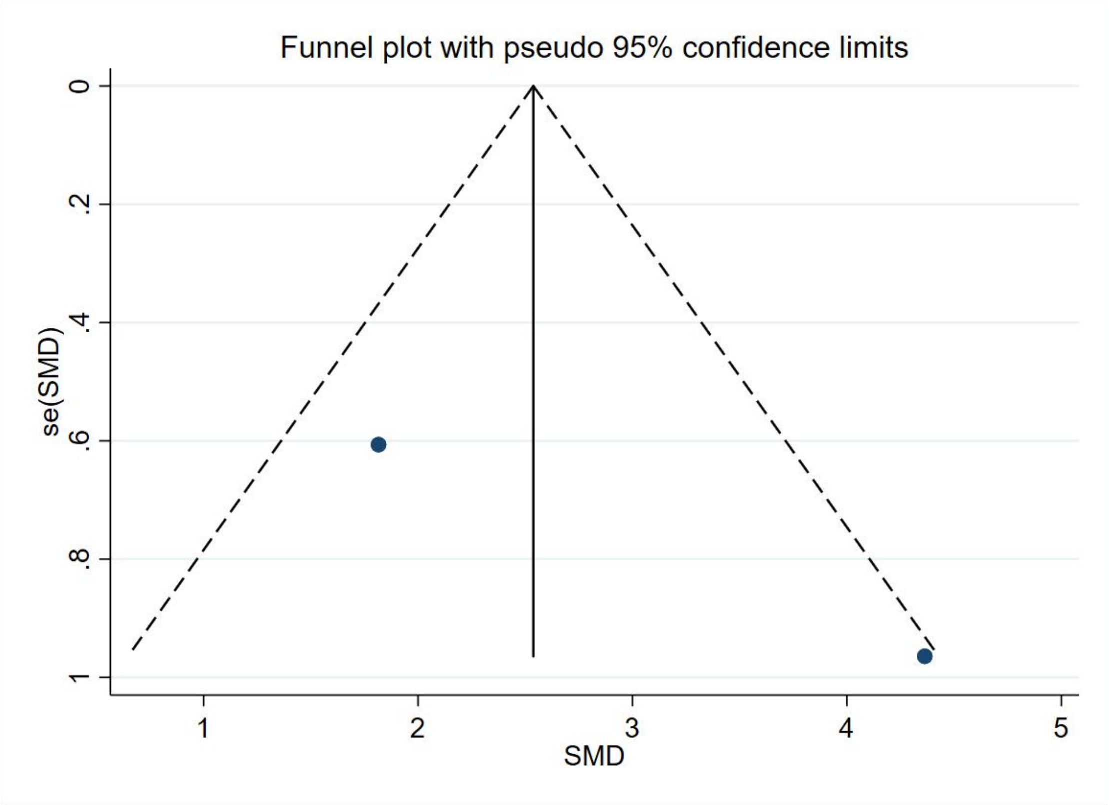

4.BDNF

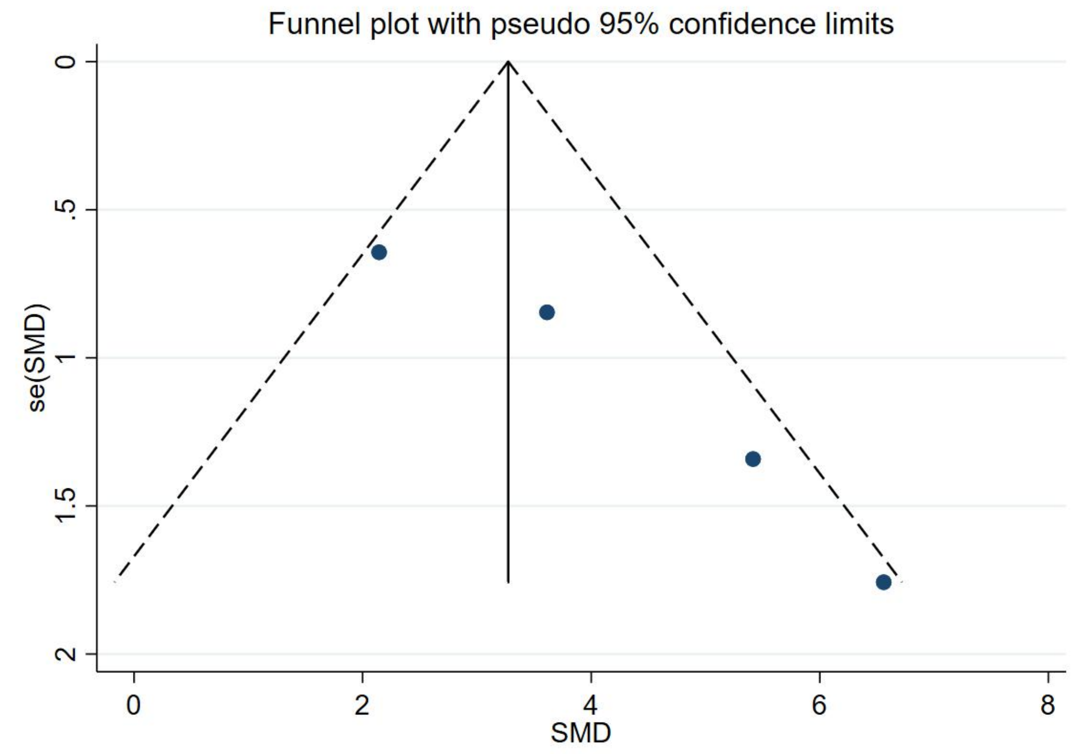

5.CREB

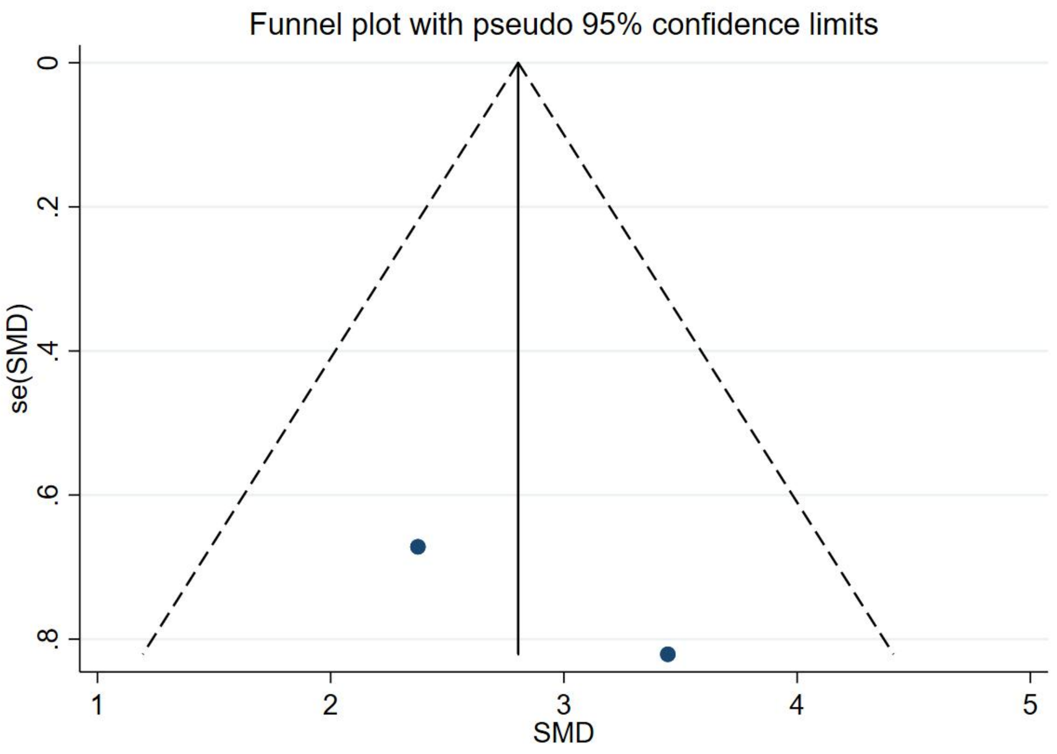

Autophagy and apoptosis factors

1.Bcl-2

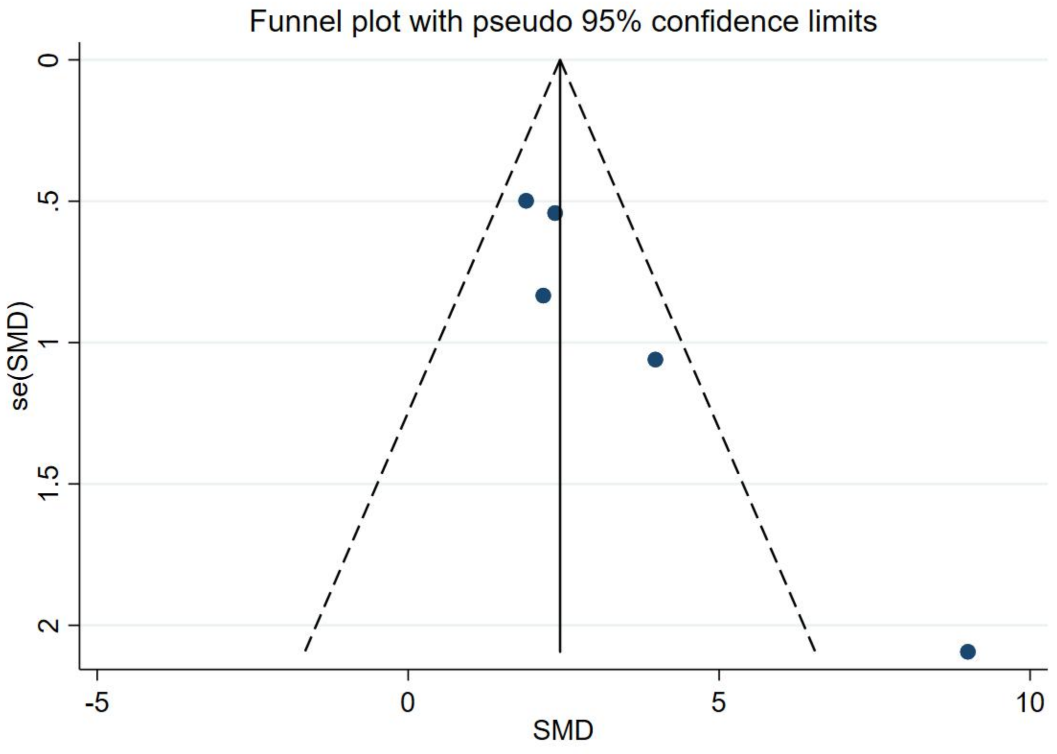

2.Beclin-1

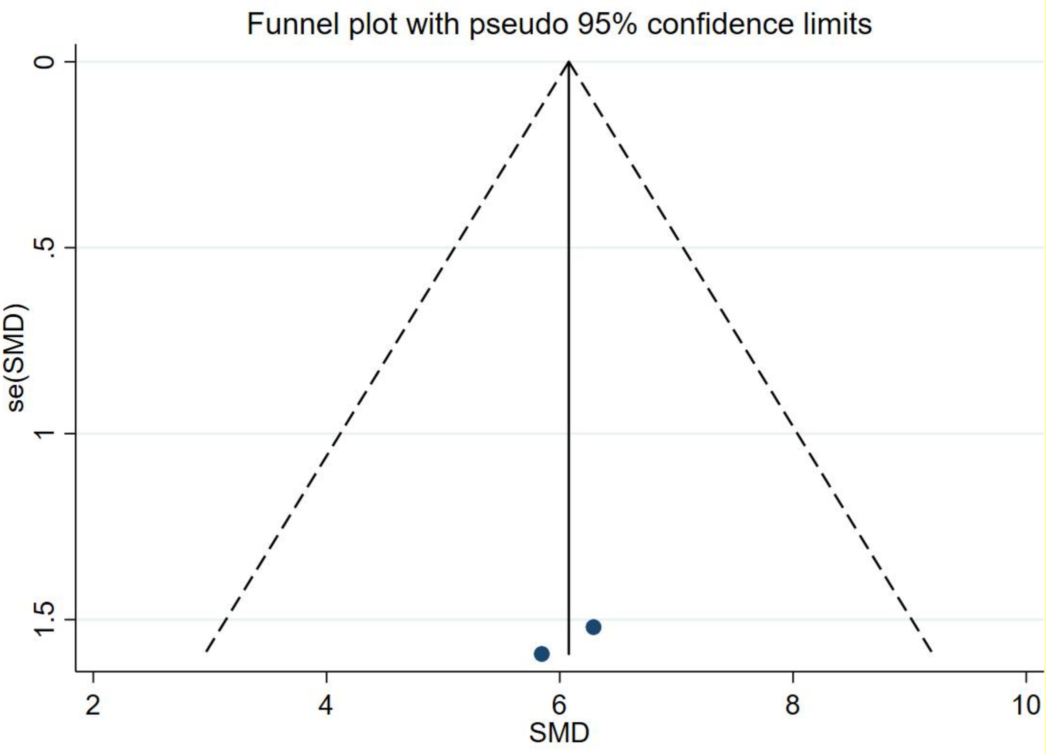

3.Caspase-3

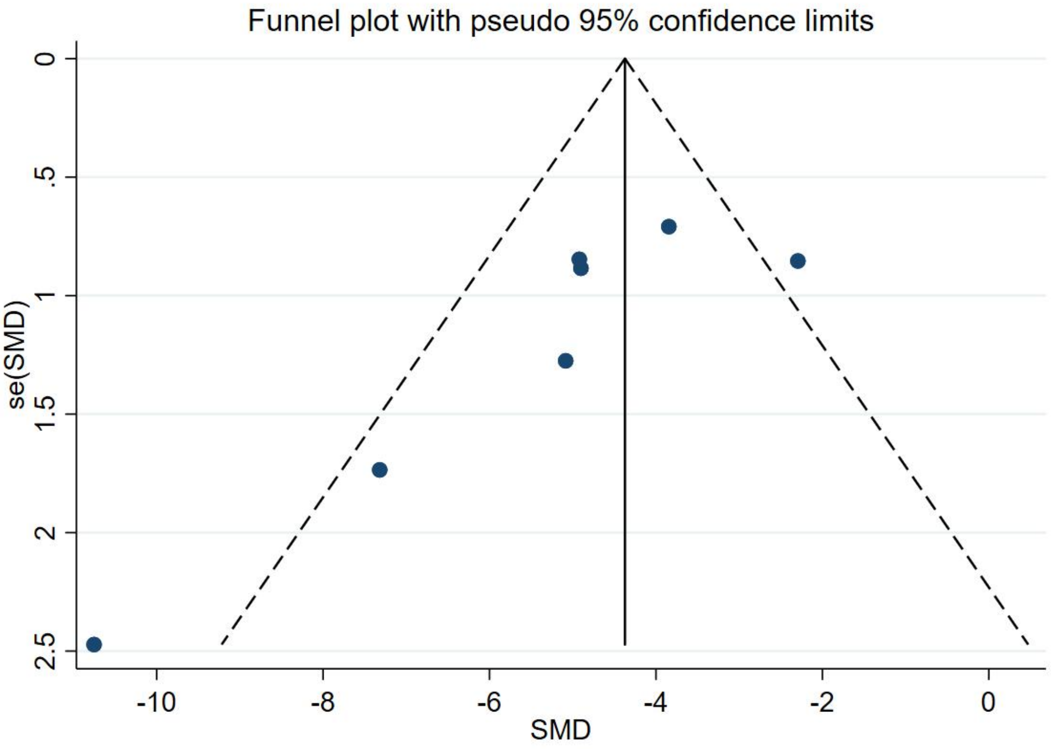

4.Apoptosis index

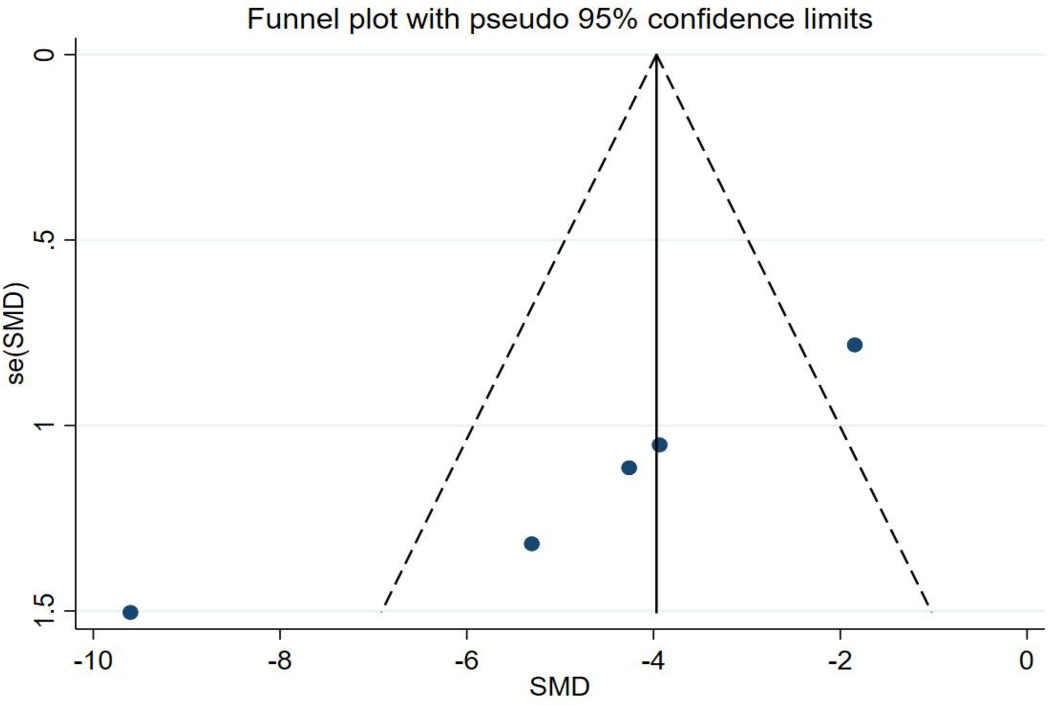

5.P62

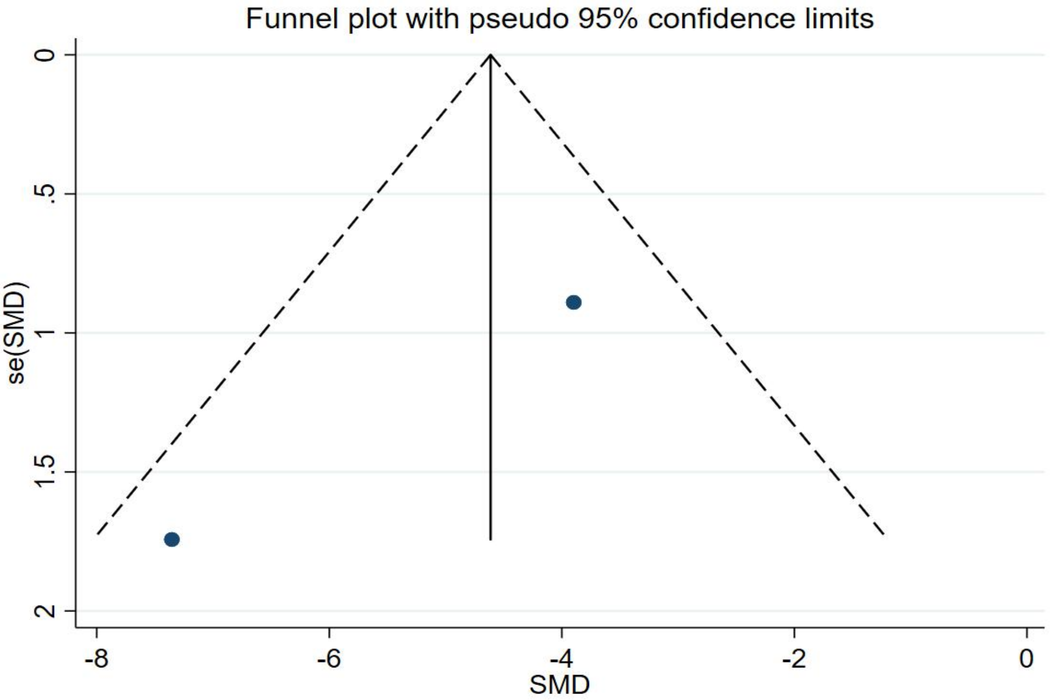

SMD, Standardized mean difference; CI, Confidence interval; IL-1β, Interleukin-1β; IL-6, Interleukin-6; TNF-α, Tumor necrosis factor-α; SOD, Superoxide dismutase; Sir2, Silent information regulator 2; GPx, Glutathione peroxidase; Nrf2, NF-E2-related factor; MDA, Malondialdehyde; 4-HNE, 4-hydroxynonenal; mNSS, Modified neurological severity score; BDNF, Brain-derived neurotrophic factor; CREB, Cyclic AMP-response element-binding protein; Bcl-2, B-cell lymphoma/leukemia 2; P62, Autophagy associated protein.
